# Initiation of Antiviral B Cell Immunity Relies on Innate Signals from Spatially Positioned NKT Cells

**DOI:** 10.1016/j.cell.2017.11.036

**Published:** 2018-01-25

**Authors:** Mauro Gaya, Patricia Barral, Marianne Burbage, Shweta Aggarwal, Beatriz Montaner, Andrew Warren Navia, Malika Aid, Carlson Tsui, Paula Maldonado, Usha Nair, Khader Ghneim, Padraic G. Fallon, Rafick-Pierre Sekaly, Dan H. Barouch, Alex K. Shalek, Andreas Bruckbauer, Jessica Strid, Facundo D. Batista

**Affiliations:** 1 Ragon Institute of MGH, MIT and Harvard, Cambridge, MA 02139, USA; 2 The Francis Crick Institute, London NW1A 1AT, UK; 3 The Peter Gorer Department of Immunobiology, King’s College London, London SE1 9RT, UK; 4 Institute for Medical Engineering & Science, MIT, Cambridge, MA 02139, USA; 5 Broad Institute, Cambridge, MA 02142, USA; 6 Center for Virology and Vaccine Research, Beth Israel Deaconess Medical Center, Harvard Medical School, Boston, MA 02215, USA; 7 Case Western Reserve University, Cleveland, OH 44106, USA; 8 Institute of Molecular Medicine, Trinity College Dublin, Dublin, Ireland; 9 Division of Immunology and Inflammation, Department of Medicine, Imperial College London, London W12 0NN, UK; 10 Department of Microbiology and Immunobiology & HMS Center for Immune Imaging, Harvard Medical School, Boston, MA 02115, USA

**Keywords:** NKT cells, B cells, macrophages, germinal center seeding, IL-4, viral infection, influenza, Zika virus, lymph node, CXCR3

## Abstract

B cells constitute an essential line of defense from pathogenic infections through the generation of class-switched antibody-secreting cells (ASCs) in germinal centers. Although this process is known to be regulated by follicular helper T (TfH) cells, the mechanism by which B cells initially seed germinal center reactions remains elusive. We found that NKT cells, a population of innate-like T lymphocytes, are critical for the induction of B cell immunity upon viral infection. The positioning of NKT cells at the interfollicular areas of lymph nodes facilitates both their direct priming by resident macrophages and the localized delivery of innate signals to antigen-experienced B cells. Indeed, NKT cells secrete an early wave of IL-4 and constitute up to 70% of the total IL-4-producing cells during the initial stages of infection. Importantly, the requirement of this innate immunity arm appears to be evolutionarily conserved because early NKT and IL-4 gene signatures also positively correlate with the levels of neutralizing antibodies in Zika-virus-infected macaques. In conclusion, our data support a model wherein a pre-TfH wave of IL-4 secreted by interfollicular NKT cells triggers the seeding of germinal center cells and serves as an innate link between viral infection and B cell immunity.

## Highlights

• NKT cells promote B cell immunity upon viral infection
• NKT cells are primed by lymph-node-resident macrophages
• NKT cells produce early IL-4 wave at the follicular borders
• Early IL-4 wave is required for efficient seeding of germinal centers

## Graphical Abstract

**Figure:**
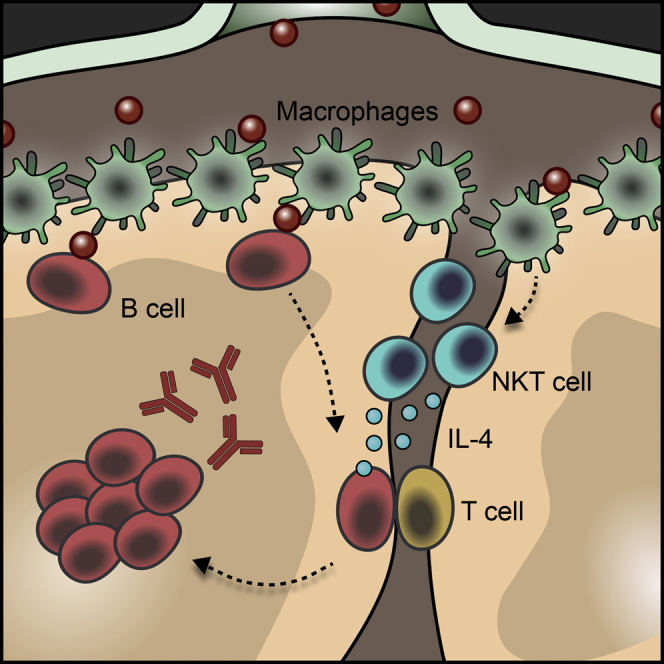

## Teaser

NKT cells are required for the initial formation of germinal centers and production of effective neutralizing antibody responses against viruses.

## Introduction

B cells are key elements of the adaptive immune response because they provide protection from pathogenic threats through the production of highly specific antibodies. In the past few years, there has been much attention on the generation of broadly neutralizing antibodies against highly variable or emerging pathogens, such as influenza, HIV, and Zika virus to develop effective vaccines capable of conferring broad and long-lasting immunity. Therefore, it is crucial to understand how viruses elicit B cell immunity during an infection process.

Pathogen recognition occurs in secondary lymphoid organs such as the spleen and lymph nodes (Batista and Harwood, 2009). In the lymph nodes, B cells recognize pathogens on the surface of subcapsular sinus (SCS) macrophages, migratory dendritic cells, and follicular dendritic cells (Carrasco and Batista, 2007, Gaya et al., 2015, Heesters et al., 2013, Junt et al., 2007, Phan et al., 2007, Qi et al., 2006). Following pathogen recognition, B cells process and present pathogen-derived peptides via major histocompatibility complex (MHC) class II molecules (Amigorena et al., 1994). This results in the recruitment of specific CD4^+^ T cells, which help the B cells to differentiate into either antibody-secreting plasma cells or germinal center (GC) cells. Within germinal centers, B cells undergo class-switch recombination and cycles of proliferation, somatic hypermutation, and affinity-based selection, finally resulting in the production of high-affinity antibodies (Victora and Nussenzweig, 2012). These processes are strictly dependent on co-stimulatory signals, mainly CD40-L, ICOS, and cytokines, such as interferon γ (IFN-γ), interleukin-4 (IL-4), and IL-21, provided by follicular helper T (TfH) cells (Weinstein et al., 2016). Because TfH cells are known to appear later during the immune response, how B cells initially seed germinal center reactions is still unclear.

In addition to pathogen-derived peptides, B cells are able to present glycolipid antigens, such as those present on the surface of *Sphingomonas* species (spp.), *Borrelia burgdorferi*, and *Streptococcus pneumoniae*, on CD1d molecules (Barral et al., 2008, Kinjo et al., 2011, Leadbetter et al., 2008). Populations of CD1d-restricted, innate-like T cells, known as natural killer (NK)T cells, recognize these lipids and provide cognate help to B cells. This NKT cell-mediated help enhances the production of antibodies by B cells (Bai et al., 2013, Barral et al., 2008, Leadbetter et al., 2008). Because there are no reports of lipid antigens originating from viruses, it is currently unclear whether NKT cells have a role in inducing antiviral B cell responses.

Here we show that, following respiratory viral infection, NKT cell-deficient mice are impaired in their ability to form germinal centers and immunoglobulin G (IgG) class-switched antibody-secreting cells (ASCs). In a striking contrast to B cell activation by glycolipid antigens, CD1d-mediated interactions between B cells and NKT cells are dispensable during viral infection. Instead, NKT cells promote B cell immunity through the early and localized production of IL-4 at the follicular borders, a process that is likely driven by CXCR3 and requires initial priming by resident macrophages through CD1d and IL-18. Importantly, this seems to be a conserved process because transcriptomic analysis of Zika-virus-infected macaques shows that early NKT and IL-4 gene signatures positively correlate with the levels of Zika-specific neutralizing antibodies. Therefore, our results indicate that the production of IL-4 by NKT cells immediately after viral infection contributes to a pre-TfH IL-4 wave at the follicular borders that facilitates the initial seeding of germinal center cells.

## Results

### NKT Cells Promote B Cell Immunity during Viral Infection

**Figure 1:**
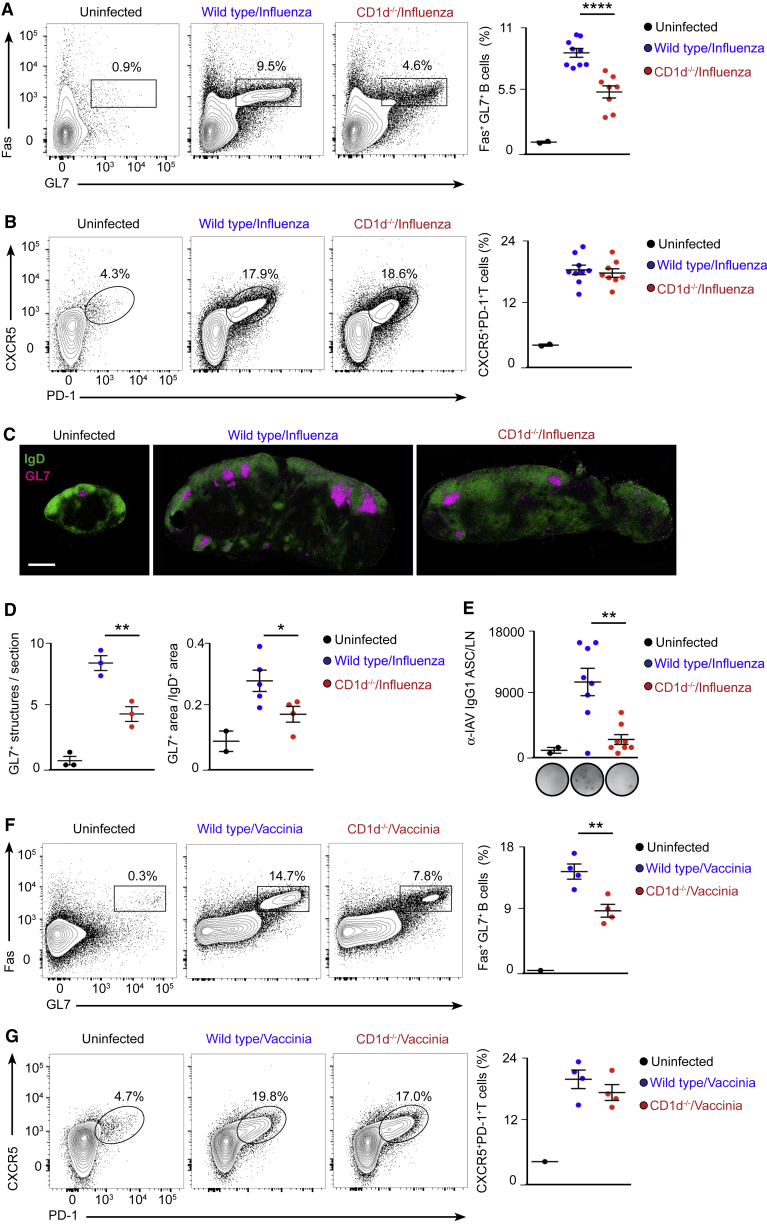
NKT Cell-Deficient Mice Exhibit Impaired Antiviral B Cell Responses (A and B) Flow cytometry analysis of mediastinal lymph nodes (LN) from control (black), wild-type (blue), or CD1d^−/−^ (red) mice on day 9 of influenza infection. Contour plots display the percentage of (A) germinal center and (B) TfH cells. (C) Confocal microscopy images of mediastinal lymph node sections from mice treated as in (A) and (B), stained with antibodies to IgD (green) and GL7 (magenta). Scale bar, 450 μm. (D) Quantification of the number and follicular area occupied by germinal centers in confocal images shown in (C) by ImageJ. (E) ELISPOT analysis of IgG1 influenza-specific ASCs in mediastinal lymph nodes from wild-type and CD1d^−/−^ mice treated as described in (A) and (B). (F and G) Flow cytometry analysis of mediastinal lymph nodes of from control (black), wild-type (blue), or CD1d^−/−^ (red) mice on day 9 of vaccinia virus infection. Contour plots display the percentage of (F) germinal center and (G) TfH cells. Data show one representative result from three experiments, where each dot represents one mouse. Data represent mean ± SEM; two-tailed paired Student’s t test, ^∗^p < 0.05, ^∗∗^p < 0.01, and ^∗∗∗∗^p < 0.0001.

**Figure S1:**
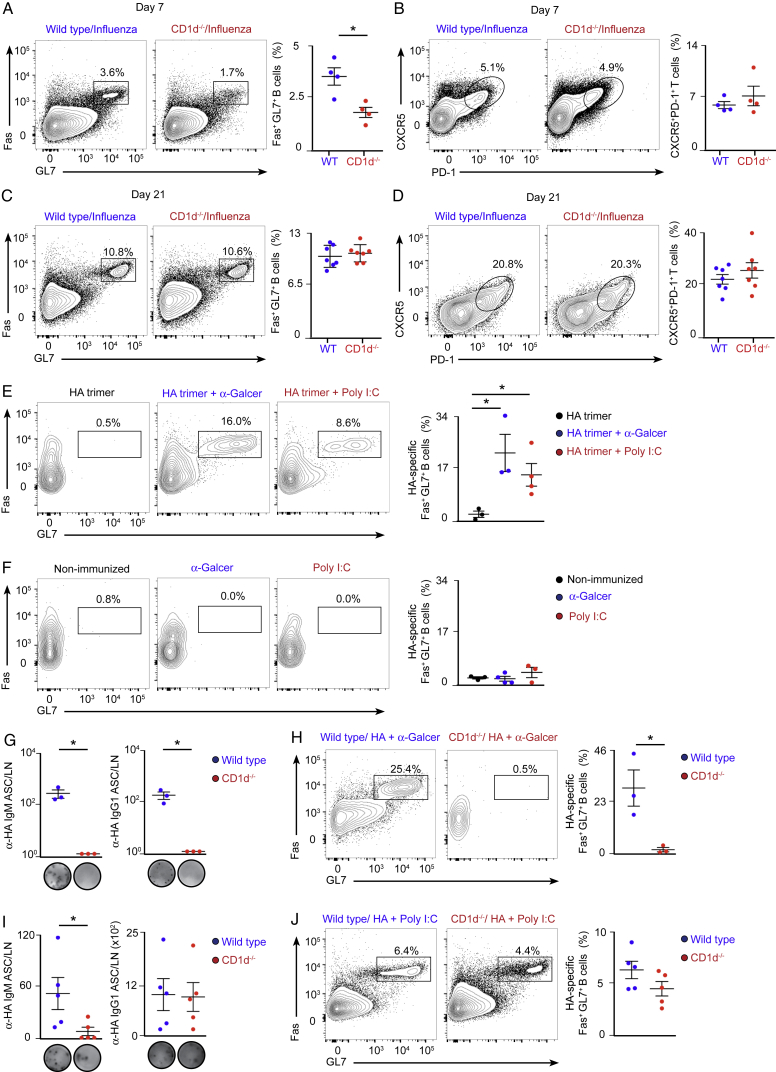
NKT Cell-Deficient Mice Display Early Impairment in B Cell Immunity, Related to Figure 1 (A–D) Flow cytometry analysis of mediastinal lymph node cells from wild type or CD1d^−/−^ mice that were intranasally infected with 200 PFU of influenza virus for (A-B) 7 or (C-D) 21 days. Representative contour plots display the percentage of (A-C) B220^+^ cells bearing biomarkers of germinal cell activity, Fas^+^GL7^+^, and (B–D) CD4^+^ T cells bearing biomarkers of TfH cells, CXCR5^+^PD-1^+^. Dot plots show quantification of (A-C) germinal center and (B-D) TfH cells. (E and F) Flow cytometry analysis of mediastinal lymph nodes harvested at day 10 from wild type mice that were intranasally challenged with (E) 10 μg of HA trimmer or (F) PBS in the presence or absence of poly I:C or α-GalCer. Representative contour plots display the percentage of HA-specific B220^+^ cells differentiated into Fas^+^GL7^+^ germinal center cells. (G and I) ELISPOT analysis of IgG1 HA-specific ASCs in mediastinal lymph nodes from wild type and CD1d^−/−^ mice treated as in figure S1E. (H and J) Flow cytometry analysis of HA-specific germinal center cells in mediastinal lymph nodes from wild type and CD1d^−/−^ mice treated as in figure S1E. In all quantification charts, each dot represents one mouse. Data are representative from two experiments. Statistical analysis two-tailed Student’s t test; ^∗^p < 0.05.

The role of NKT cells in inducing the humoral response to glycolipid-bearing antigens has been established (Bai et al., 2013, Barral et al., 2008, Leadbetter et al., 2008); however, because virus-derived lipid antigens are unknown, we sought to investigate the role of NKT cells in the induction of B cell responses during viral infections. Accordingly, we intranasally infected C57BL/6 wild-type and CD1d^−/−^ mice, which lack CD1d and type I and II NKT cells, with 200 plaque-forming units (PFUs) of influenza A virus, Puerto Rico/8/34 (PR8) strain. After 7, 9, and 21 days of infection, lung-draining lymph nodes were harvested, and adaptive immune response development was assessed by flow cytometry. We observed a progressive expansion of Fas^+^GL7^+^ germinal center cells and CXCR5^+^PD-1^+^ TfH cells during the course of infection in wild-type animals compared with uninfected ones (Figures 1A and 1B; Figures S1A–S1D). Interestingly, the formation of germinal center cells was significantly reduced in NKT cell-deficient mice at early time points, whereas the expansion TfH cells remained similar throughout the infection process (Figures 1A and 1B; Figures S1A–S1D). We further compared the spatial organization of germinal centers in wild-type and CD1d^−/−^ mice by immunohistochemistry. In wild-type animals, influenza infection induces both the enlargement of IgD^+^ B cell follicles as well as the formation of follicular GL7^+^ structures corresponding to germinal centers (Figure 1C). In contrast, in NKT cell-deficient animals, germinal center structures were reduced by day 9 of infection, a defect that was accompanied by a strong decrease in the generation of influenza-specific IgG1 ASCs (Figures 1C–1E). Interestingly, CD1d^−/−^ mice also displayed a significant reduction in germinal center formation when infected with the vaccinia virus Western Reserve strain, suggesting that the ability of NKT cells to promote B cell responses is a general feature of viral infections (Figures 1F and 1G).

To address whether NKT cells would also induce B cell immunity after vaccination with viral antigens, we intranasally immunized wild-type and CD1d^−/−^ mice with hemagglutinin (HA) trimmer from the PR8 influenza strain in the presence or absence of two different adjuvants: alpha-galactosylceramide (α-GalCer) or poly(I:C). We found that HA-specific germinal centers developed in lung-draining lymph nodes only when HA was administered together with either of these adjuvants (Figures S1E and S1F). Interestingly, the formation of HA-specific germinal centers and ASCs is completely abrogated in NKT-cell-deficient mice when α-GalCer was co-administered, demonstrating that NKT cells are essential to induce B cell immunity when exogenous glycolipids are used as adjuvants (Figures S1G and S1H). In addition, when poly(I:C) was co-administered, we observed a diminution in the number of IgM ASCs and a consistent, although non-significant, reduction in HA-specific germinal centers in CD1d^−/−^ animals. These results indicate that NKT cells may also enhance B cell immunity during intranasal vaccination in the absence of exogenous glycolipids (Figures S1I and S1J).

### NKT Cells Deliver Non-cognate Help to B Cells to Induce Antiviral Immunity

**Figure 2:**
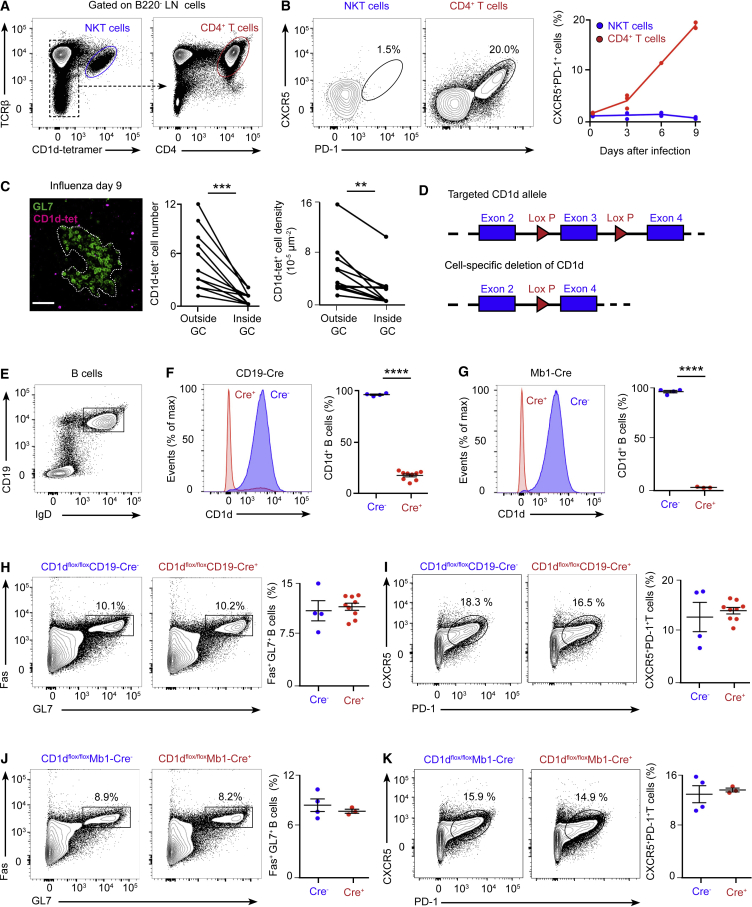
NKT Cells Deliver Non-cognate Help to B Cells during Viral Infection (A and B) Flow cytometry analysis of wild-type mice infected with influenza virus. (A) Gating strategy for NKT and CD4^+^ T cells. B220^+^ cells were excluded from the analysis. (B) Analysis of the percentage of NKT (blue) and CD4^+^ T (red) cells that acquire CXCR5 and PD-1 markers after infection. (C) Confocal microscopy analysis of lymph nodes on day 9 of influenza infection incubated with CD1d tetramer (magenta) and GL7 (green). Charts show the number and density of NKT cells inside and outside of germinal centers. Each dot represents a single germinal center. Scale bar, 60 μm. (D) Schematic showing the CD1d locus targeted for deletion with two loxP sites flanking exon 3 of CD1d. (E) Representative contour plot shows the gating strategy for B cells. (F and G) Flow cytometry analysis of CD1d expression in B cells from (F) CD1d^flox/flox^CD19-Cre or (G) CD1d^flox/flox^Mb1-Cre mice: Cre^−^ (blue) and Cre^+^ (red). (H–K) Flow cytometry analysis of germinal center and TfH cell formation in (H and I) CD1d^flox/flox^CD19-Cre or (J and K) CD1d^flox/flox^Mb1-Cre mice on day 9 of influenza infection: Cre^−^ (blue dots) and Cre^+^ (red dots). All data are representative of 3 independent experiments. Unless specified, each dot represents one mouse. Horizontal bars, mean; error bars, SEM; statistical analysis, two tailed Student’s t test, ^∗∗^p < 0.01, ^∗∗∗^p < 0.001, and ^∗∗∗∗^p < 0.0001.

**Figure S2:**
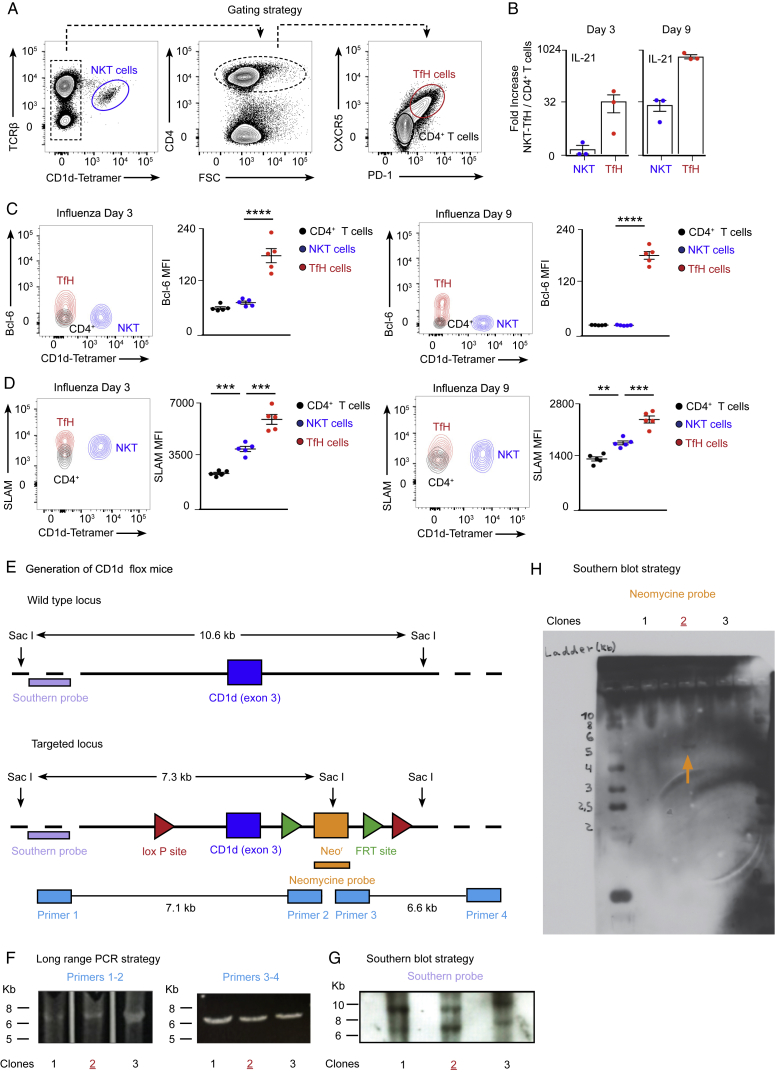
Expression Analysis of TfH Markers by NKT Cells and Strategy for the Generation of CD1d-Floxed ESCs, Related to Figure 2 (A) Flow cytometry gating strategy for the analysis of NKT cells (TCRβ^+^ CD1d tetramer^+^), CD4^+^ T cells (TCRβ^+^ CD1d tetramer^-^ CD4^+^) and TfH cells (TCRβ^+^ CD1d tetramer^-^ CD4^+^ CXCR5^+^ PD-1^+^) in mediastinal lymph nodes at day 9 of influenza infection. (B) RT-qPCR analysis of IL-21 expression at day 3 and 9 of influenza infection by sorted NKT and TfH cells compared to CD4^+^ cells. (C and D) Representative contour plots show the expression of (C) Bcl-6 and (D) SLAM by NKT, TfH and CD4^+^ T cells at day 3 and 9 of infection. Quantification on the right charts shows the mean fluorescence intensity for (C) Bcl-6 and (D) SLAM. (E) Scheme showing the wild type and targeted CD1d locus containing two loxP sites flanking the exon 3 of CD1d as well as two FRT sites for the removal of the Neomycin resistance cassette. (F) Specific fragments amplified by long-range PCR for the 5′ end (7.1kb) and 3′ end (6.6kb) of the clones that correctly performed homologous recombination. (G) DNA from three positive clones was digested with SacI restriction enzyme and Southern blot performed using a labeled Southern probe. The upper band corresponds to the wild type locus (10.6kb) and the lower band to the targeted locus (7.3kb). (H) Southern blot of the three positive clones performed with a labeled Southern probe against the Neomycin cassette. Orange arrow indicates the single 5.2kb band recognized by this probe, indicating single insertion of the construct. The clone 2, in red, was chosen for injections. In all quantification charts, each dot represents one mouse. Data are representative from three experiments. Statistical analysis two-tailed Student’s t test; ^∗∗^p < 0.01, ^∗∗∗^p < 0.001 and ^∗∗∗∗^p < 0.0001.

Having shown that CD1d^−/−^ mice have diminished B cell responses during influenza and vaccinia virus infection, we sought to determine the mechanism by which NKT cells help B cells to mount an antiviral immune response. In the case of bacterium-derived glycolipids, NKT cells differentiate into CXCR5^+^PD-1^+^Bcl6^+^ follicular helper NKT (NKTfH) cells, which engage in CD1d-mediated cognate interactions with B cells, produce IL-21, and support germinal center responses (Chang et al., 2011, King et al., 2011). To address whether NKT cells differentiate into NKTfH cells after viral infection, wild-type mice were intranasally infected with influenza virus, and mediastinal lymph nodes were harvested after 0–9 days. Flow cytometry analysis revealed that, although conventional CD4^+^ T cells gradually express CXCR5, PD-1, Bcl6, and SLAM and produce high levels of IL-21 after infection, TCRβ^+^CD1d-tetramer^+^ NKT cells do not upregulate Bcl6, CXCR5, or PD-1 and express moderate levels of SLAM and IL-21 (Figures 2A and 2B; Figures S2A–S2D). These results indicate that NKT cells do not acquire NKTfH cell phenotype after viral infection.

To further explore the localization of NKT cells within the tissues of infected mice, we stained mediastinal lymph nodes of wild-type mice with R-phycoerythrin-labeled, PBS-57-loaded CD1d-tetramer as reported previously (Lee et al., 2015). Lymph node sections were further stained for GL7 and analyzed by confocal microscopy. We found that tetramer-labeled cells localized in the periphery of GL7^+^ areas, whereas almost no tetramer-labeled cells were found inside these GL7^+^ areas (Figure 2C). This observation indicates that NKT cells are excluded from the germinal center after viral infection.

To directly address whether CD1d-mediated cognate interaction between B cells and NKT cells is required for the development of the antiviral B cell response, we generated CD1d^flox/flox^ mice to delete CD1d specifically on B cells. To this end, two loxP sites were inserted, flanking exon 3 of CD1d, and CD1d^flox/flox^ mice were then crossed with mice expressing the Cre recombinase under the promoter of CD19 or Mb1 genes (Figure 2D; Figures S2E–S2H). Deletion of CD1d was observed in approximately 80% of B cells in CD1d^flox/flox^CD19-Cre^+^ mice and in 100% of B cells in CD1d^flox/flox^Mb1-Cre^+^ mice, indicating that the Mb1-Cre system is a more efficient tool to delete CD1d on B cells (Figures 2E–2G). Then, groups of CD1d^flox/flox^CD19-Cre^+^ and CD1d^flox/flox^Mb1-Cre^+^ and their corresponding Cre^−^ littermates were intranasally infected with influenza virus, and mediastinal lymph nodes were harvested after 9 days. The formation of germinal centers and TfH cells was similar in CD1d^flox/flox^ CD19-Cre^−^ and CD19-Cre^+^ mice and in CD1d^flox/flox^ Mb1-Cre^−^ and Mb1-Cre^+^ animals, indicating that CD1d-mediated interactions between B cells and NKT cells are not required for the development of germinal centers during viral infection (Figures 2H–2K).

### An Early NKT Cell Wave of IL-4 Precedes Late IL-4 Production by TfH Cells

**Figure 3:**
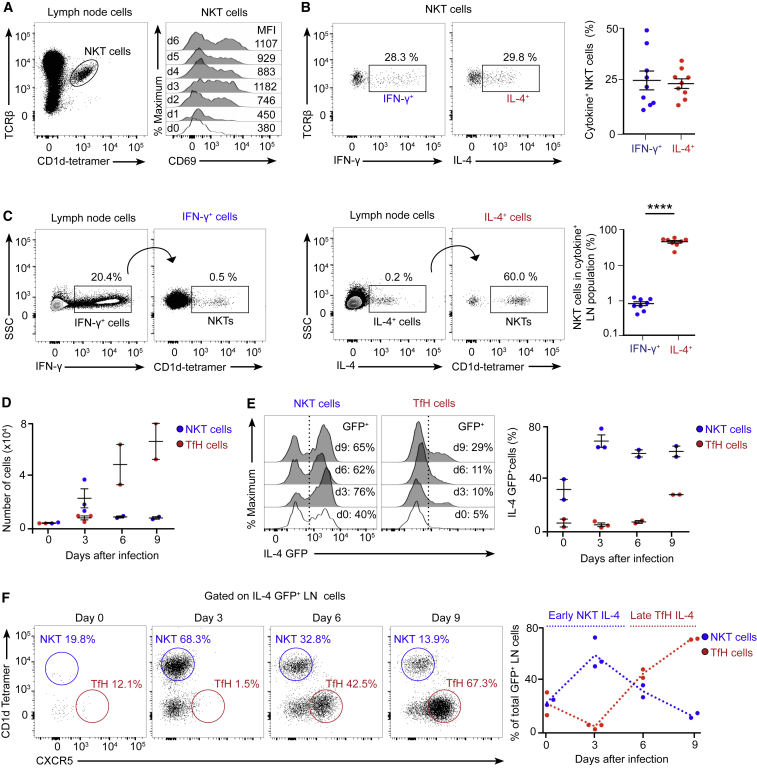
NKT Cells Generate a Pre-TfH Cell Wave of IL-4 (A) Flow cytometry analysis of CD69 levels in the TCRβ^+^ CD1d tetramer^+^ population after 0–6 days of influenza infection. MFI, mean fluorescence intensity. (B) Representative plots showing the percentage of NKT cells producing IFN-γ (blue) and IL-4 (red) after 3 days of influenza infection. (C) Flow cytometry analysis of IFN-γ (blue) and IL-4 (red) production on day 3 of influenza infection. Gates were drawn on IFN-γ^+^ and IL-4^+^ lymph node cells to analyze the percentage of NKT cells inside each cytokine^+^ population. (D–F) Flow cytometry analysis of NKT (blue) and TfH (red) cells from IL-4 GFP mice infected with influenza virus. (D) Numbers of NKT and TfH cells at different stages of influenza infection. (E) GFP expression in NKT and TfH cells at different times of infection. (F) Percentage of CD1d-tetramer^+^ NKT cells and CXCR5^+^ TfH cells inside the IL-4 GFP^+^ lymph node cell population at different stages of influenza infection. In all charts, each dot represents one mouse. In graphs showing statistical analysis, data are representative of three independent experiments. Horizontal bars, mean; error bars, SEM. Student’s t test, ^∗∗∗∗^p < 0.0001.

**Figure S3:**
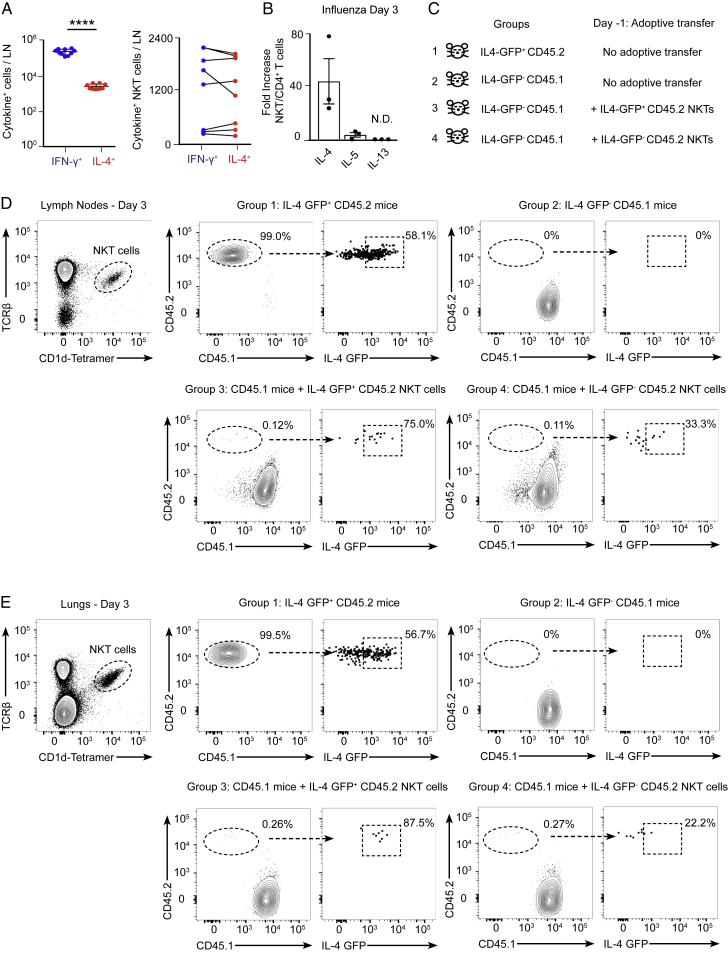
Analysis of the NKT Cell Source of IL-4, Related to Figure 3 (A) Quantification of IFN-γ^+^ (blue) and IL-4^+^ (red) producing lymph node (left chart) or NKT cells (right chart) in mediastinal lymph nodes after 3 days of influenza infection. (B) RT-qPCR analysis of IL-4, IL-5 and IL-13 expression at day 3 of influenza infection by sorted NKT cells compared to CD4^+^ T cells. Each dot represents one mouse. (C) Adoptive transfer strategy for the analysis of the source of IL-4^+^ NKT cells after influenza infection. (D and E) Flow cytometry analysis at day 3 of influenza infection of (D) mediastinal lymph node or (E) lung NKT cells from mice that were treated as in (C) to detect IL4-GFP expression. Data are representative from two experiments.

One mechanism by which NKT cells might initiate antiviral B cell immunity in a non-cognate manner is likely to be via the production of B cell-modulatory cytokines. To examine the ability of NKT cells to produce these molecules, we first assessed their kinetics of activation after influenza infection by measuring the levels of the surface activation marker CD69. We observed that, although most NKT cells were negative for CD69 in uninfected mice (day 0), the number of CD69^+^ NKT cells peaked 3 days after infection, indicating that activated NKT cells accumulated early after viral challenge (Figure 3A). Interestingly, influenza infection not only results in an upregulation of activation markers but also in a robust production of IFN-γ and IL-4, with a similar percentage of NKT cells producing either cytokine (Figure 3B; Figure S3A). Notably, even though NKT cells produce IFN-γ after infection, they represent only 1% of the total IFN-γ^+^ cell population in lymph nodes (Figure 3C). In striking contrast, NKT cells represent up to 50%–70% of the total IL-4^+^ cells after influenza infection (Figure 3C); however, we did not detect NKT cell production of other type 2 cytokines, such as IL-5 and IL-13 (Figure S3B). These observations demonstrate that, although the population of NKT cells is much smaller than those of conventional T cells, they represent the main source of IL-4 in lymph nodes at the early stages of influenza infection.

Previous studies have shown that TfH cells constitute essentially all of the IL-4-producing T cells in draining lymph nodes after infection (King and Mohrs, 2009, Reinhardt et al., 2009, Yusuf et al., 2010). However, these cells usually accumulate later during the infection process. To gain insight into the kinetics of IL-4 production by NKT and TfH cells after influenza infection, we took advantage of IL-4 GFP reporter mice, which concomitantly express endogenous levels of IL-4 in addition to GFP (Mohrs et al., 2001). Flow cytometry analysis revealed an accumulation of NKT cells shortly after influenza infection, reaching a maximum number on day 3, and a gradual but continuous accumulation of TfH cells, reaching a maximum number on day 9 (Figure 3D). In agreement with previous studies, we observed GFP expression in approximately 20%–40% of NKT cells even on day 0, suggesting that NKT cells already produce IL-4 mRNAs at steady state (Figure 3E; Lee et al., 2013, Mohrs et al., 2005). However, the percentage of GFP^+^ NKT cells peaks up to 60% to 80% after 3 days of infection, indicating that influenza infection rapidly induces IL-4 production by NKT cells (Figure 3E). Notably, both steady-state IL-4 producers and those that acquire the *de novo* ability to produce IL-4 can contribute to the early pool of IL-4 secretors (Figures S3C–S3E). In contrast, the percentage of GFP^+^ TfH cells increases gradually after infection, with around 10% of GFP^+^ TfH cells on day 3 and 30% of GFP-expressing TfH cells on day 9 of infection (Figure 3E). These results indicate that, although NKT cells accumulate and become IL-4 producers rapidly after infection, TfH cells differentiate and produce IL-4 later throughout the infection process.

To assess the contribution of NKT and TfH cells to the pool of IL-4-producing cells at different times of infection, we gated on TCRβ^+^ IL-4-GFP^+^ lymph node cells and analyzed the proportion of this population that was CD1d-tetamer^+^ (NKT cells) or CXCR5^+^ (TfH cells). Interestingly, we observed that, 3 days after influenza infection, almost 70% of GFP^+^ cells were NKT cells, whereas less than 2% were TfH cells (Figure 3F). This trend is reversed around 6 days after infection, and, by day 9, less than 15% of GFP^+^ cells were NKT cells, whereas almost 70% were TfH cells (Figure 3F). These results indicate that, during influenza infection, there is an early wave of IL-4, in which NKT cells constitute the main source of this cytokine, and a late wave of IL-4, where TfH cells overcome NKT cells as the main IL-4 producers.

### An Early NKT Cell Wave of IL-4 Occurs at the Follicular Borders

**Figure 4:**
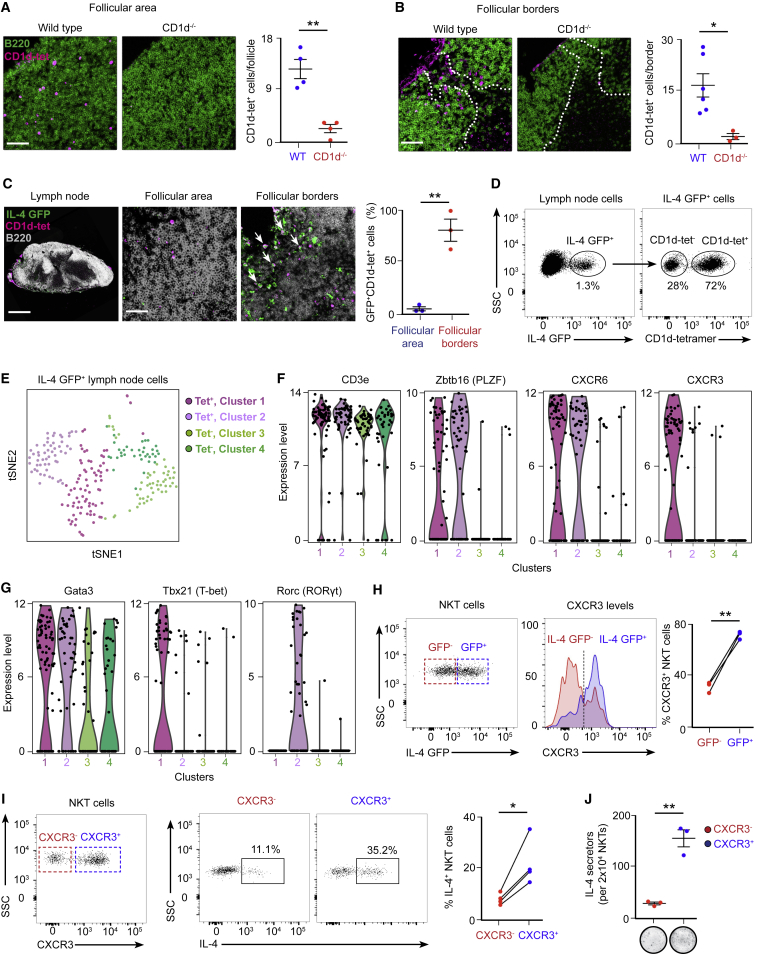
The Early IL-4 Wave Is Localized at the Periphery of B Cell Follicles (A–C) Confocal microscopy analysis of (A and B) wild-type and CD1d^−/−^ and (C) IL4-GFP mice on day 3 of influenza infection. Lymph nodes were labeled with CD1d tetramer (magenta) and anti-B220 antibody (green in A and B and white in C). The arrows in (C) indicate CD1d tetramer^+^ cells expressing IL-4 (IL-4 GFP, green). Scale bars, 300 μm (lymph node) and 60 μm (section). (D) Flow cytometry analysis of IL-4 GFP^+^ cells in mediastinal lymph nodes on day 3 of influenza infection, showing CD1d-tet^−^ and CD1d-tet^+^ cells. (E) t-SNE plots of CD1d-tet^−^ and CD1d-tet^+^ subsets. The CD1d-tet^+^ population separates into two clusters (1, dark purple; 2, light purple), as does the CD1d-tet^−^ population (3, light green; 4, dark green). (F and G) Expression distribution (violin plots) in each population (horizontal axes) for (F) CD3e, Zbtb16, CXCR6, and CXCR3 and (G) Gata3, Tbx21, and Rorc. Expression levels are log_2_(tpm+1). (H) Flow cytometry analysis of CXCR3 levels in IL-4-GFP^−^ (red) and GFP^+^ (blue) NKT cells on day 3 of influenza infection. Lines connect cells from the same mouse. (I) Flow cytometry analysis of IL-4 production by CXCR3^−^ (red) and CXCR3^+^ (blue) NKT cells on day 3 of influenza infection. Lines connect cells from the same mouse. (J) ELISPOT analysis of IL-4-producing CXCR3^−^ (red) and CXCR3^+^ (blue) NKT cells sorted on day 3 of influenza infection. Each dot represents pooled NKT cells from 6 mice. In graphs showing statistical analysis, data are representative of at least two independent experiments. Horizontal bars, mean; error bars, SEM. Student’s t test, ^∗^p < 0.05, ^∗∗^p < 0.01.

**Figure S4:**
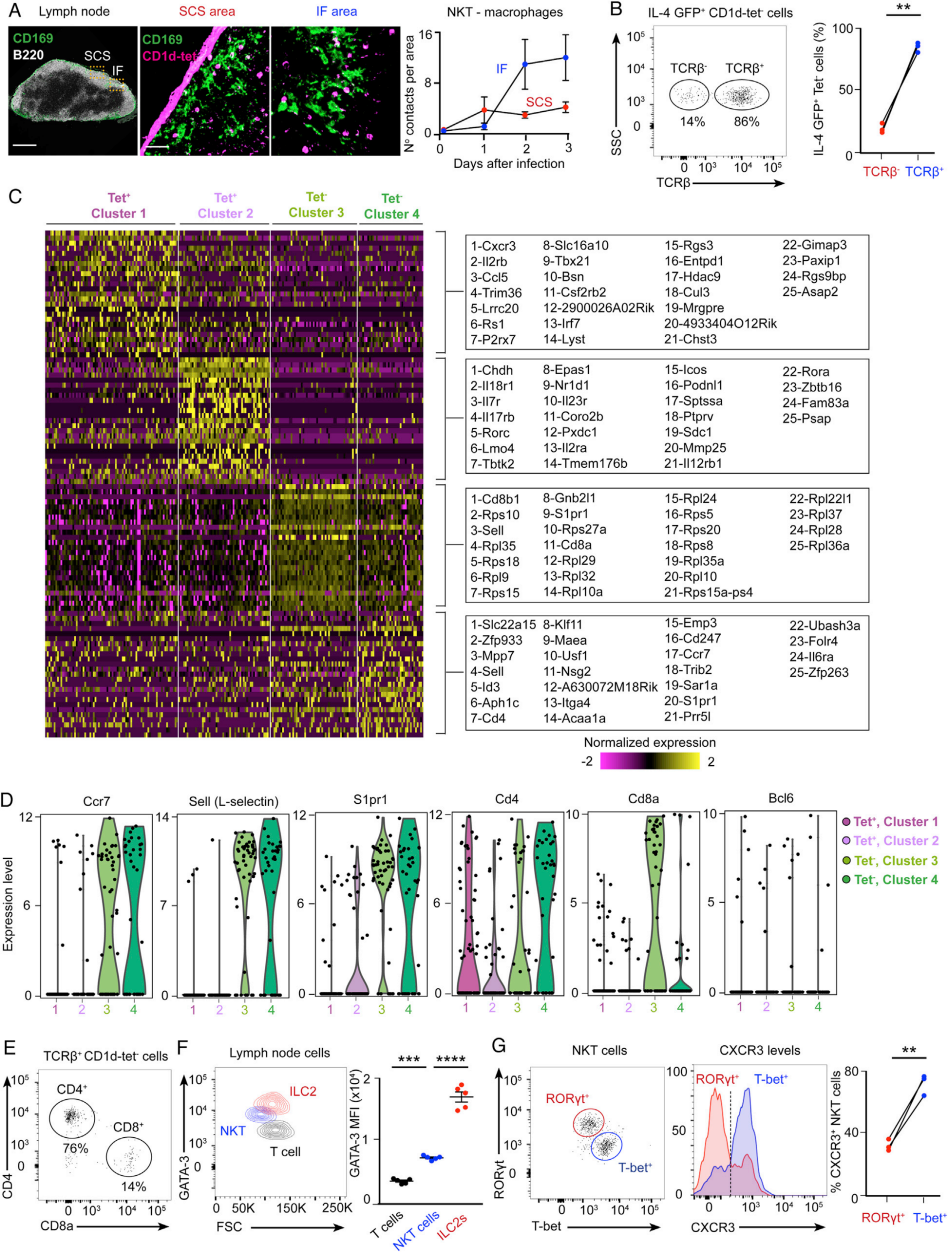
Single-Cell RNA-Seq Analysis of IL-4^+^ Lymph Node Cells, Related to Figure 4 (A) Confocal microscopy of mediastinal lymph nodes sections at day 3 of influenza infection. Lymph nodes were labeled with PBS-57 loaded CD1d tetramer (magenta) and antibodies to B220 (white) and CD169 (green). IF, interfollicular. Scale bars, 300 μm (left); 60 μm (right). Chart on the right shows the quantification of NKT cells-macrophages contacts observed in the different areas of the lymph node at day 0, 1, 2 and 3 of infection in mediastinal lymph nodes. Data are representative from three experiments. (B) Flow cytometry analysis of IL4-GFP^+^ CD1d-tet^-^ cells in mediastinal lymph nodes at day 3 of influenza infection showing TCRβ^-^ and TCRβ ^+^ cells. (C) Single-cell RNA-seq heatmap showing the expression of 25 significant marker genes (Table S1) for the CD1d-tet^+^ clusters (columns 1 and 2) and CD1d-tet^-^ clusters (columns 3 and 4). Single cells and genes were determined on the basis of unsupervised hierarchical clustering (Table S1). (D) Expression distribution (violin plots) in each population (horizontal axes) for Ccr7, Sell, S1pr1, CD4, CD8a and Bcl6. Expression levels are Log_2_(tpm+1). (E) Flow cytometry analysis of IL4-GFP^+^ TCRβ^+^ CD1d-tet^-^ cells in mediastinal lymph nodes at day 3 of influenza infection showing CD4^+^ and CD8a ^+^ cells. (F) Representative contour plot show the expression of GATA-3 by NKT cells, ILC2s (Live/Dead^-^ B220^-^ CD3^-^ CD5^-^ CD11b^-^ CD11c^-^ CD127^+^ CD45^+^ GATA-3^+^) and CD4^+^ T cells at day 3 of infection. Quantification on the right chart shows the mean fluorescence intensity for GATA-3. (G) Flow cytometry analysis of RORγt^+^ and T-bet^+^ NKT cells in mediastinal lymph nodes at day 3 of influenza infection. Histogram shows the levels of CXCR3 in RORγt^+^ and T-bet^+^ populations. Dot graphs show the quantification of CXCR3^+^ NKT cells in the RORγt^+^ (red dots) and T-bet^+^ populations (blue dots), with lines connecting NKT cells from the same mouse. In graphs showing statistical analysis, data are representative of at least two independent experiments. Statistical analysis two-tailed Student’s t test; ^∗∗^p < 0.01, ^∗∗∗^p < 0.001 and ^∗∗∗∗^p < 0.0001.

So far, we have defined the temporal frame of IL-4 production by NKT cells during the early stages of influenza infection. To gain insight into the spatial distribution of these IL-4-producing NKT cells, we infected wild-type, CD1d^−/−^, and IL-4 GFP reporter mice with influenza virus and harvested mediastinal lymph nodes after infection. The lymph nodes were incubated with labeled PBS-57-loaded CD1d-tetramer, and sections were further stained against B220 and CD169, a macrophage marker, and analyzed by confocal microscopy. Although CD1d-tetramer^+^ cells were nearly undetectable in uninfected animals, they were observed inside B cell follicles and in direct contact with CD169^+^ macrophages at the subcapsular sinus and interfollicular areas by day 3 of infection (Figures 4A and 4B; Figure S4A). In contrast, CD1d-tetramer^+^ cells were almost absent in lymph nodes from CD1d^−/−^ animals, indicating that CD1d-tetramer^+^ cells are most likely NKT cells (Figures 4A and 4B). Interestingly, although the great majority of CD1d-tetramer^+^ cells located inside the B cell follicles do not express GFP, most of the GFP^+^ cells appear to be located in the areas surrounding B cell follicles (Figure 4C). These results indicate that early IL-4 production is restricted to the periphery of the B cell follicles, where antigen-specific B cells relocate to recruit T cell help after activation.

To get insight into the extent of cellular heterogeneity within the immune cells contributing to the early IL-4 wave, we characterized the IL-4 GFP^+^ population by flow cytometry 3 days post-infection with influenza virus. This experiment shows that approximately 70% of this population corresponds to NKT cells (CD1d-tet^+^), whereas the remaining 30% (CD1d-tet^−^) are mostly T cells (TCRβ^+^), excluding a significant contribution of ILC2s to the early wave of IL-4 (Figure 4D; Figure S4B). To further study these two cell populations, IL-4 GFP^+^ CD1d-tet^+^ and IL-4 GFP^+^ CD1d-tet^−^ cells were single cell-sorted and analyzed by RNA sequencing (RNA-seq). After processing (STAR Methods), we retained 222 cells from the CDd1-tet^+^ (n = 113) and CDd1-tet^−^ (n = 109) populations in our single-cell RNA-seq analysis. T-distributed stochastic neighbor embedding (t-SNE) and shared nearest neighbor (SNN) clustering revealed that single cells from the two subsets (CD1d-tet^+^ and CD1d-tet^−^) each bifurcate into two separate clusters, reflecting divergent transcriptional states within these populations (Figures 4E; Figure S4C; Table S1). Independent of the cluster, most of these cells express CD3e, confirming their T cell origin (Figure 4F). A feature of the CD1d-tet^−^ subset clusters is the expression of CCR7, L-selectin, and S1PR1, mRNAs encoding proteins that control T cell migration (Figure S4D). Furthermore, one of the CD1d-tet^−^ clusters is characterized by cells expressing high levels of CD4 and the other one by cells expressing CD8a. Importantly, only a small proportion of the cells in these two clusters express Bcl-6, ruling out a significant contribution of TfH cells to this early IL-4 wave (Figures 3F; Figures S4D and S4E). On the other hand, significant numbers of cells from the CD1d-tet^+^ subset express PLZF, a transcription factor that directs the NKT cell effector program (Figure 4F). Interestingly, a large proportion of cells from both CD1d-tet^+^ clusters express the chemokine receptor CXCR6, whereas only one of them expresses CXCR3 (Figure 4F). Both CXCR3^+^ and CXCR3^−^ clusters express GATA-3, whereas the CXCR3^+^ group specifically expresses T-bet, and the CXCR3^−^ one expresses RORγt, an observation that was further confirmed by flow cytometry (Figures 4G; Figures S4F and S4G).

CXCR3 has been reported to recruit T cells to the interfollicular zones, the areas where we find most of the IL-4 GFP^+^ NKT cells after infection (Groom et al., 2012, Sung et al., 2012). Interestingly, flow cytometry analysis of lymph nodes from influenza-infected mice revealed that 80% of IL-4 GFP^+^ NKT cells express CXCR3, whereas only 30% of IL-4 GFP^−^ NKT cells express this marker (Figure 4H). Therefore, we wondered whether CXCR3^−^ and CXCR3^+^ NKT cells would have a differential ability to produce IL-4 after infection. To this end, we analyzed IL-4 production by both CXCR3^−^ and CXCR3^+^ NKT cell groups on day 3 of influenza infection by flow cytometry and enzyme-linked immunospot (ELISPOT). Strikingly, the number of cells secreting IL-4 in the CXCR3^+^ NKT cell sub-population is five times higher than in the CXCR3^−^ group (Figures 4I and 4J). These results suggest that CXCR3 preferentially drives the recruitment of IL-4-producing NKT cells to interfollicular areas.

Taken together, our results indicate that influenza infection triggers two spatiotemporally divergent waves of IL-4: an early wave, mainly produced by NKT cells and restricted to the periphery of B cell follicles, and a late one, produced by germinal center-resident TfH cells.

### Resident Macrophages Trigger an Early NKT Cell Wave of IL-4 and B Cell Immunity

**Figure 5:**
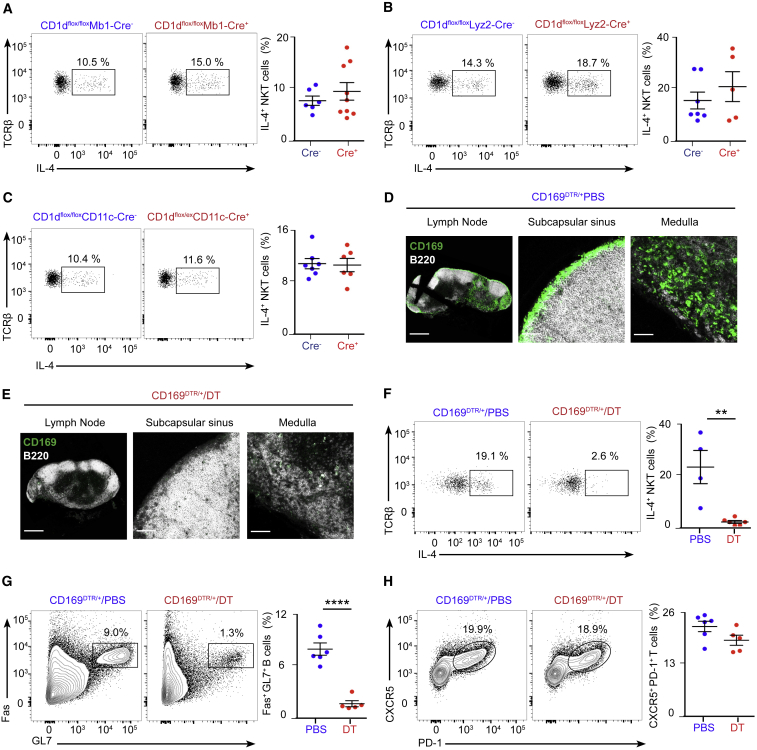
Resident Macrophages Promote Early IL-4 Production by NKT Cells and Antiviral B Cell Immunity (A–C) Flow cytometry analysis of IL-4 production by NKT cells in (A) CD1d^flox/flox^Mb1-Cre, (B) CD1d^flox/flox^Lyz2-Cre, and (C) CD1d^flox/ex^CD11c-Cre mice on day 3 of influenza infection: Cre^−^ (blue dots) and Cre^+^ (red dots). (D and E) Confocal microscopy analysis of CD169^DTR/+^ mice after 4 days of (D) PBS or (E) diphtheria toxin (DT) administration. Sections were stained with antibodies to B220 (white) and CD169 (green). Scale bars, 300 μm (left) and 60 μm (center and right). (F) Flow cytometry analysis of IL-4 production by NKT cells from CD169^DTR/+^/PBS-treated or CD169^DTR/+^/DT-treated mice on day 3 of influenza infection: PBS (blue dots) and DT (red dots). (G and H) Flow cytometry analysis of germinal center (G) and TfH (H) cells in CD169^DTR/+^/PBS-treated and CD169^DTR/+^/DT-treated mice on day 9 of influenza infection: PBS (blue dots) and DT (red dots). In all panels, each dot represents one mouse. Data show one representative result from three experiments. Data represent mean ± SEM; two-tailed paired Student’s t test, ^∗∗^p < 0.01, ^∗∗∗∗^p < 0.0001.

**Figure S5:**
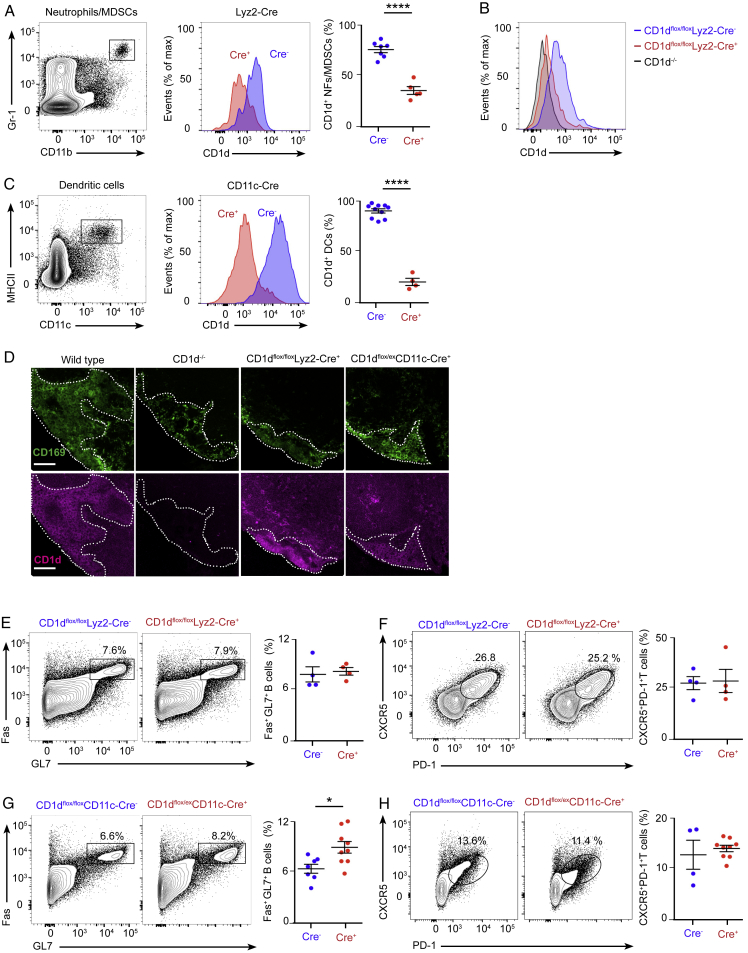
Conditional Targeting of CD1d in the Myeloid Lineage, Related to Figure 5 (A–C) Flow cytometry plots showing the gating strategy for (A) Gr-1^+^CD11b^+^ neutrophils (NFs) and myeloid-derived suppressor cells (MDSCs) and (C) MHCII^+^CD11c^+^ dendritic cells (DCs). Red and blue histograms depict the levels of CD1d in the gated populations in (A) CD1d^flox/flox^Lyz2-Cre^-^ versus Cre^+^ and (B) CD1d^−/−^ mice or (C) CD1d^flox/ex^CD11c-Cre^-^ versus Cre^+^ mice. These quantitative differences are depicted via dot plots; blue and red dots show CD1d^+^ cells in the Cre^-^ and Cre^+^ animals, respectively. (D) Confocal microscopy of mediastinal lymph nodes sections from wild type, CD1d^−/−^, CD1d^flox/flox^Lyz2-Cre^+^ and CD1d^flox/ex^CD11c-Cre mice. Sections were stained with antibodies to CD169 (green) and CD1d (magenta). Scale bars, 60 μm. (E–H) Flow cytometry analysis of mediastinal lymph node cells at day 9 of influenza infection. Representative contour plots show the percentage of B cells and T cells from (E-F) CD1d^flox/flox^Lyz2-Cre^-^ and Cre^+^ or (G-H) CD1d^flox/ex^CD11c-Cre^-^ and Cre^+^ mice that acquire (E and G) germinal center markers, Fas and GL7, or (F and H) TfH cell markers, CXCR5 and PD-1. Quantifications are shown in dot plots, Cre^-^ mice (blue dots) and Cre^+^ mice (red dots). In all panels, each dot represents a single mouse. Horizontal bars – mean; error bars – SEM. Data are representative of three independent experiments. Statistical analysis, two tailed Student’s t test, ^∗^p < 0.05, ^∗∗∗∗^p < 0.0001.

**Figure S6:**
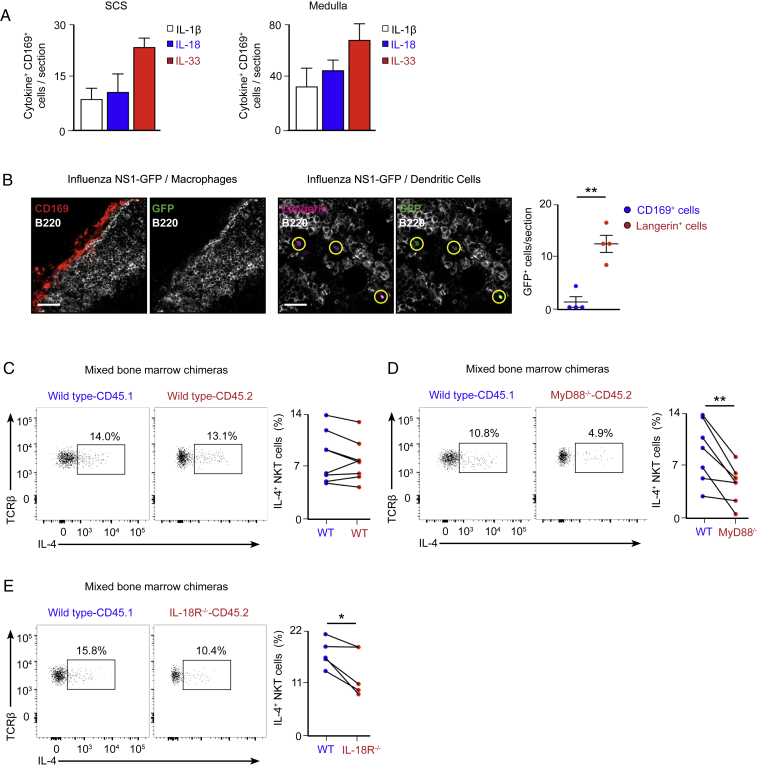
Intrinsic Role of MyD88 and IL-18R in NKT Cell Production of IL-4, Related to Figure 6 (A) Quantification of IL-1β^+^, IL-18^+^ and IL-33^+^ cells in the SCS and medullar areas from the images shown in Figures 6C and 6D. (B) Confocal microscopy of mediastinal lymph nodes from wild type mice that were infected with 200 PFU of PR8 NS1-GFP and sacrificed after 3 days. Sections were stained with antibodies to CD169 (red), B220 (gray) and Langerin (magenta). Scale bars, 60 μm. Quantification of GFP^+^ cells is shown in the right chart. (C–E) Flow cytometry analysis of chimeric mice that were generated by transferring a 1:1 bone marrow mixture of wild type (CD45.1^+^) and (C) wild type (CD45.2^+^), (D) MyD88^−/−^ (CD45.2^+^) or (E) IL-18R^−/−^ (CD45.2^+^) to sub-lethally γ-irradiated CD45.1 recipient mice. Eight weeks after bone marrow transplant, mice were infected with 200 PFU of influenza virus and mediastinal lymph nodes were harvested after further 3 days. Flow cytometry panels show the production of IL-4 by CD45.1^+^ and CD45.2^+^ NKT cells in the different sets of chimeras. Dot graphs show the quantification of IL-4^+^ NKT cells in the CD45.1^+^ population (blue dots) and the CD45.2^+^ population (red dots), with lines connecting the two NKT cell subpopulation coming from the same chimera. In all panels, each dot represents a single mouse. Horizontal bars – mean; error bars – SEM. Data are representative of two independent experiments. Statistical analysis, two tailed Student’s t test, ^∗^p < 0.05, ^∗∗^p < 0.01.

**Figure S7:**
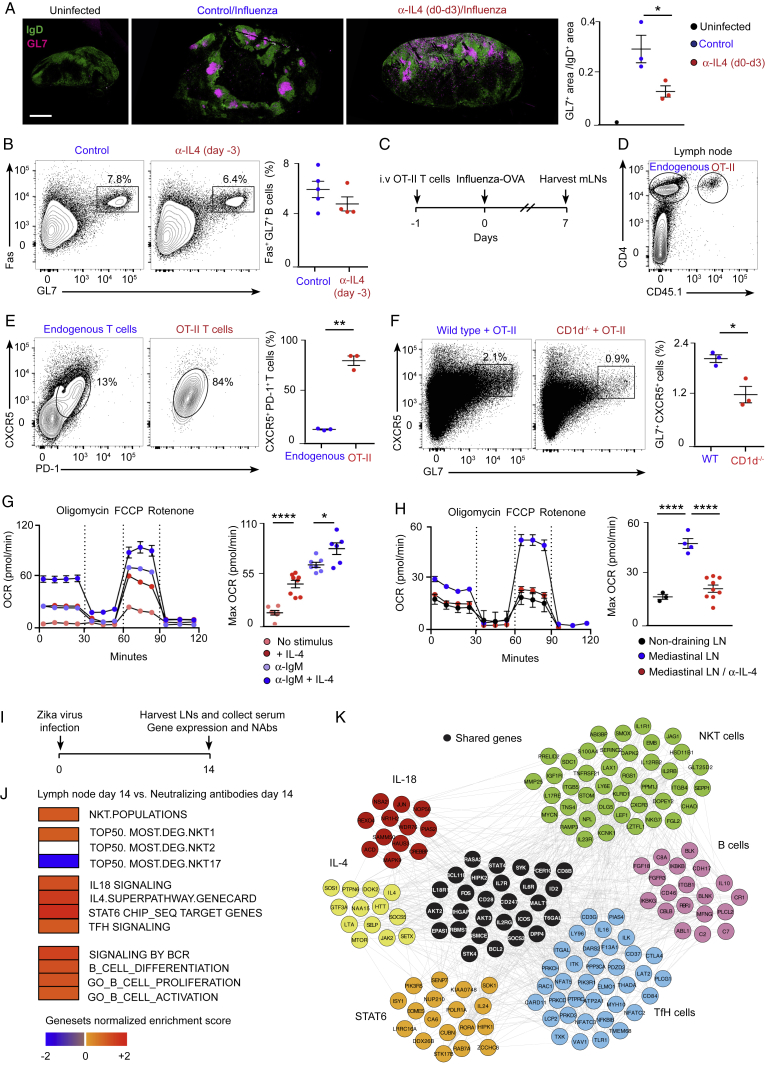
Importance of Early IL-4 Production on B Cell Immunity, Related to Figure 7 (A) Representative confocal microscopy images of mediastinal lymph node sections from mice that were treated as in Figure 7E. Sections were stained with antibodies to the B lymphocyte marker, IgD (green) and the germinal center marker, GL7 (magenta). Scale bar, 450 μm. Quantification of the germinal center area in confocal images by ImageJ is shown in the right chart. (B) Flow cytometry analysis at day 8 of mediastinal lymph nodes from wild type mice that were treated with PBS or anti-IL4 blocking antibody on day −3, and infected with 200 PFU of influenza virus at day 0. Contour plots show Fas^+^GL7^+^ germinal center cells. The responses are quantified in right charts. (C) Experimental scheme showing the strategy for the generation of antigen-specific TfH cell precursors. (D–F) Flow cytometry analysis of mediastinal lymph node cells from mice treated as in (C). Representative contour plots display (D) the gating strategy used to differentiate endogenous CD4^+^ T cells from adoptively transferred OT-II cells, (E) the percentage of endogenous and exogenous cells bearing the TfH cell markers PD-1 and CXCR5 and (F) the percentage of B cells that acquire GL7 marker. Quantifications are shown on the right charts. (G and H) OCR at baseline and after sequential treatment with oligomycin, FCCP and Rotenone of B cells that were (G) stimulated *ex vivo* with anti-IgM and/or IL-4 or (H) isolated from lymph node cells of influenza-infected mice (day 3). Bar charts show the respiratory capacity obtained as (OCR after FCCP)- (basal OCR). Each dot represents an individual measurement. (I) Experimental scheme showing Zika virus infection strategy and time points at which lymph nodes and serum were collected from macaques. (J) Linear regression model of gene signatures for NKT cells, TfH cells, B cells, IL-4, STAT6 and IL-18 from harvested lymph nodes at day 14 with neutralizing antibodies measured in plasma at day 17. Red squares represent positive correlation, blue squares represent negative correlation and white squares represent no significant correlation. (K) Gene interacting network inference using GeneMANIA software representing interconnectivity of NKT, TfH cells, B cells, IL-4, STAT6 and IL-18 pathways and the co-expression of their leading genes. Unless stated, each dot represents a single mouse. Horizontal bars – mean; error bars – SEM. Data are representative of two independent experiments. Statistical analysis, two tailed Student’s t test, ^∗^p < 0.05, ^∗∗^p < 0.01 and ^∗∗∗∗^p < 0.0001.

To define the immune machinery involved in the induction of this early NKT cell-derived IL-4, we specifically abrogated CD1d expression on different immune cell populations by crossing our CD1d^flox/flox^ mice with animals expressing the Cre recombinase under the control of the Mb1, Lyz2, or CD11c gene promoters. Flow cytometry analysis showed an effective deletion of CD1d on Gr-1^+^CD11b^+^ neutrophils/myeloid-derived suppressor cells (MDSCs) in CD1d^flox/flox^Lyz2-Cre^+^ mice and on MHCII^+^CD11c^+^ dendritic cells in CD1d^flox/ex^CD11c-Cre^+^ mice (Figures S5A–S5C). However, no deletion of CD1d could be obtained in lymph node CD169^+^ macrophages in these transgenic lines (Figure S5D). Groups of CD1d^flox/flox^Mb1-Cre^+^, CD1d^flox/flox^Lyz2-Cre^+^, and CD1d^flox/ex^CD11c-Cre^+^ and their corresponding Cre^−^ littermates were intranasally infected with influenza virus, and mediastinal lymph nodes were harvested after 3 or 9 days. Interestingly, similar percentages of IL-4^+^ NKT cells (day 3) and germinal centers and TfH cells (day 9) were observed among these transgenic lines (Figures 2H–2K and 5A–5C; Figures S5E–58H). These results indicate that CD1d-mediated cognate interactions between NKT cells and B cells, neutrophils/MDSCs, or dendritic cells are not required for NKT cell-mediated induction of IL-4 production or germinal center formation.

Because we were unable to delete CD1d on CD169^+^ macrophages by using CD11c or Lyz2 Cre lines, we decided to take a macrophage depletion approach to assess the importance of these macrophages in the induction of IL-4 production by NKT cells. Accordingly, mice expressing the diphtheria toxin receptor (DTR) under the CD169 promoter, here named CD169-diphtheria toxin receptor mice, were treated with PBS or diphtheria toxin. Although PBS injection has no effect on macrophages, a single injection of diphtheria toxin is sufficient to deplete both subcapsular sinus and medullar macrophages from lymph nodes (Figures 5D and 5E). After 4 days of PBS or diphtheria toxin administration, mice were intranasally infected with influenza virus, and mediastinal lymph nodes were harvested 3 or 9 days later. Interestingly, as revealed by flow cytometry, the production of IL-4 by NKT cells (day 3) as well as the formation of germinal centers (day 9) were severely impaired in macrophage-depleted mice (Figures 5F and 5G). Notably however, the development of TfH cells remained unaltered in the absence of macrophages (Figure 5H). These results indicate that CD169^+^ macrophages are essential for early triggering of NKT cell effector programs and the initiation of B cell response following viral infection.

### Induction of NKT Cell-Derived IL-4 by Macrophages Requires CD1d and IL-18

**Figure 6:**
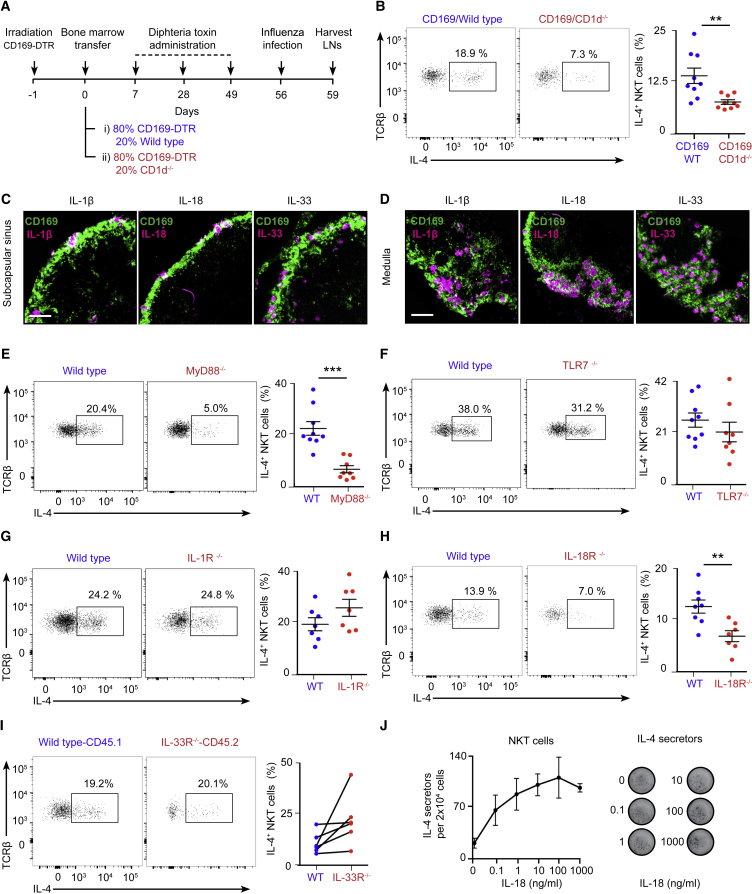
Resident Macrophages Promote Early NKT Cell IL-4 Production through CD1d and IL-18 (A) Experimental scheme showing the strategy for the generation of chimeras lacking CD1d specifically on CD169^+^ macrophages. (B) Flow cytometry analysis of IL-4 production by NKT cells from mice subjected to the experimental scheme show in (A): wild-type chimeras (blue dots) and CD1d^−/−^ chimeras (red dots). (C and D) Confocal microscopy analysis on day 2 of influenza infection showing (C) SCS and (D) medullar areas. Sections were stained with antibodies to CD169 (green) and IL-1β/IL-18/IL-33 (magenta). Scale bars, 60 μm. (E–I) Flow cytometry analysis of wild-type and (E) MyD88^−/−^, (F) TLR7^−/−^, (G) IL-1R^−/−^, and (H) IL-18R^−/−^ mice or (I) IL-33R^−/−^ chimeric mice on day 3 of influenza infection. Charts show IL-4^+^ NKT cells in wild-type (blue dots) or mutant (red dots) populations. (J) ELISPOT analysis of IL-4 production by sorted NKT cells incubated *ex vivo* with IL-18. Each dot represents one mouse. Data show mean ± SEM and are representative of 3 independent experiments. Statistical analysis: two-tailed Student’s t test, ^∗∗^p < 0.01, ^∗∗∗^p < 0.001.

Because the depletion of CD169^+^ macrophages abolished the production of IL-4 by NKT cells, we investigated whether CD1d-mediated interactions between CD169^+^ macrophages and NKT cells are required for NKT cell activation. To address this question, we generated mixed bone marrow chimeras lacking CD1d expression specifically on CD169^+^ macrophages. For this, CD169-DTR mice were sub-lethally irradiated and, the following day, adoptively transferred with either 80% CD169-DTR cells + 20% wild-type cells or 80% CD169-DTR cells + 20% CD1d^−/−^ cells. After 1, 4, and 7 weeks, mice were treated with diphtheria toxin, and, at week 8 of reconstitution, both groups of chimeras were intranasally infected with influenza virus (Figure 6A). Although we observed a robust production of IL-4 in CD169-DTR/wild-type chimeras by day 3 of infection, NKT cells from CD169-DTR/CD1d^−/−^ chimeras were impaired regarding this cytokine (Figure 6B). This result demonstrates that close interactions between CD169^+^ macrophages and NKT cells through CD1d are necessary to induce IL-4 production by NKT cells after viral infection.

The depletion of CD169^+^ macrophages has a more profound effect on the induction of IL-4 production by NKT cells than solely the deletion of CD1d on these cells. Therefore, we wondered whether CD169^+^ macrophages could enhance IL-4 synthesis through the secretion of co-stimulatory cytokines. To address this question, we stained lymph nodes on day 2 of influenza infection with antibodies against CD169, IL-1β, IL-18, and IL-33. These cytokines are secreted early after infection and have been shown to modulate the differentiation and function of innate and adaptive lymphoid cells (Kastenmüller et al., 2012, Sagoo et al., 2016). We detected the accumulation of IL-1β, IL-18, and IL-33 in both subcapsular sinus and medullar macrophages, suggesting that these resident macrophages are a source of inflammatory cytokines (Figures 6C and 6D; Figure S6A). Interestingly, their production is not the consequence of direct infection of macrophages because no viral replication was detected on them when a recombinant influenza virus carrying a GFP reporter gene was used (Manicassamy et al., 2010; Figure S6B).

Next we tested whether macrophage-derived IL-1β, IL-18, or IL-33 are required to induce NKT cell production of IL-4. These three cytokines are known to signal through the MyD88 adaptor protein (Adachi et al., 1998, Schmitz et al., 2005); hence, as a first approach, we compared the ability of wild-type versus MyD88^−/−^ NKT cells to produce IL-4 after influenza infection. Our flow cytometry results show that, although a substantial proportion of lymph node NKT cells from wild-type mice produce IL-4, NKT cells from MyD88^−/−^ animals are severely impaired in the production of this cytokine (Figure 6E). Importantly, their impairment is not due to lack of Toll-like receptor (TLR) signaling because NKT cells from TLR7^−/−^ mice secrete IL-4 to the same extent as NKT cells from wild-type animals (Figure 6F). We then explored whether signaling through IL-1R, IL-18R, or IL-33R is involved in this process. We performed influenza infection in wild-type, IL-1R^−/−^, or IL-18R^−/−^ mice and mixed bone marrow chimeras transferred with 50% CD45.1 wild-type and 50% CD45.2 IL-33R^−/−^ cells. We found that IL-1R and IL-33R were not required for IL-4 production because NKT cells from IL-1R^−/−^ mice and IL-33R^−/−^ experimental chimeras did not show an impairment in IL-4 production compared with their wild-type counterparts (Figures 6G–6I). In contrast, the percentage of NKT cells producing IL-4 was significantly reduced in IL-18R^−/−^ mice, suggesting that IL-18 enhances IL-4 secretion by NKT cells (Figure 6H). Remarkably, MyD88 and IL-18R were intrinsically required by NKT cells because MyD88^−/−^ and IL-18R^−/−^ NKT cells displayed a reduced ability to produce IL-4 compared with wild-type cells in a mixed bone marrow chimera setup (Figures S6C–S6E). Furthermore, IL-18 could induce IL-4 production on itself when added *ex vivo* to primary NKT cells (Figure 6J). Altogether, our results indicate that the IL-18R-MyD88 axis plays an important role in the production of IL-4 by NKT cells.

### An Early IL-4 Wave Initiates B Cell Immunity to Viral Infection

**Figure 7:**
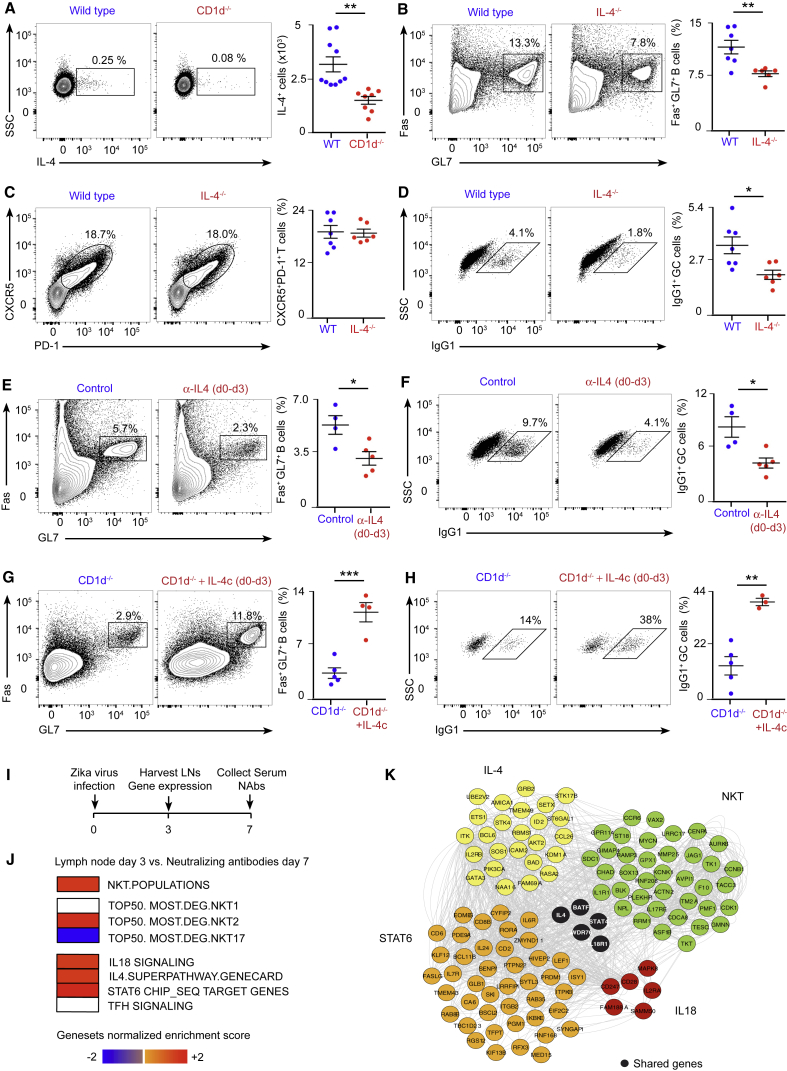
A Conserved Early IL-4 Wave Is Critical for Induction of B Cell Immunity (A) Flow cytometry analysis on day 3 of influenza infection. Graphs show the number of IL-4^+^ lymph node cells in wild-type (blue dots) and CD1d^−/−^ (red dots) animals. (B–D) Flow cytometry analysis on day 8 of influenza infection. Graphs show the percentage of (B) germinal center, (C) TfH, and (D) IgG1^+^ cells in wild-type (blue dots) and IL-4^−/−^ mice (red dots). (E and F) Flow cytometry analysis on day 8 of influenza infection of wild-type mice treated with PBS or blocking α-IL-4 antibody during the initial 3 days of infection. Graphs show (E) germinal center and (F) IgG1^+^ cells in wild-type (blue dots) and α-IL-4 treated mice (red dots). (G and H) Flow cytometry analysis on day 8 of influenza infection of CD1d^−/−^ mice treated with PBS or IL-4 complex (IL-4c) during the initial 3 days of infection. Graphs show (G) germinal center cells and (H) IgG1^+^ cells in wild-type (blue dots) and IL-4c-treated mice (red dots). (I) Experimental scheme showing the Zika virus infection strategy. (J) Linear regression model of gene signatures for NKT cells, TfH cells, IL-4, STAT6, and IL-18 from harvested lymph nodes on day 3 with neutralizing antibodies measured in plasma on day 7. Red squares, positive correlation; blue squares, negative correlation; white squares, no significant correlation. (K) Gene-interacting network inference representing interconnectivity of NKT cells, IL-4, STAT6, and IL-18 pathways. In (A)–(H), each dot represents a single mouse. Data are representative of three independent experiments. Statistical analysis: two-tailed Student’s t test, ^∗^p < 0.05, ^∗∗^p < 0.01, ^∗∗∗^p < 0.001.

Because of the central role of NKT cells in producing IL-4 early after infection, we wondered whether CD1d^−/−^ mice would have reduced numbers of IL-4-secreting cells. To address this question, wild-type and CD1d^−/−^ mice were infected with influenza virus, and the amount of IL-4^+^ cells was measured on day 3. The number of cells secreting IL-4 was significantly reduced in CD1d^−/−^ compared with wild-type animals, indicating that other immune cells cannot compensate for the absence of NKT cells (Figure 7A). Next, to test whether the impaired B cell responses observed in CD1d^−/−^ animals could be due to reduced IL-4 production, FVB wild-type and IL-4^−/−^ mice were infected with influenza virus, and, 8 days later, mediastinal lymph nodes were harvested. A substantial expansion of germinal center and TfH cells was observed in wild-type mice compared with uninfected animals (Figures 7B and 7C). Interestingly, in IL-4^−/−^ mice, there was a significant reduction in the proportion of germinal centers, whereas the differentiation of TfH cells remained similar to that of wild-type animals (Figures 7B and 7C). Furthermore, germinal center cells from IL-4^−/−^ mice have a reduced capacity to class-switch to IgG1 (Figure 7D). These results show that IL-4 is necessary for the induction of the B cell response after influenza infection.

Then we asked whether the early wave of IL-4 was necessary for the induction of B cell responses after influenza infection. To address this question, wild-type mice were infected with influenza virus, and a sub-group of them was treated with a blocking IL-4 antibody during the first 3 days of infection. By day 8, we observed a significant reduction in the formation of germinal centers and in their ability to class-switch to IgG1 in mice treated with anti-IL-4 (Figures 7E and 7F; Figure S7A). Importantly, when we injected anti-IL-4 3 days prior to infection, this reduction was not significant, suggesting that the blocking effect of this antibody only lasts for a few days (Figures S7B). Altogether, these results suggest that the early IL-4 wave is necessary to promote B cell immunity.

Subsequently, to check whether the recovery of the initial IL-4 wave could rescue the germinal center defect observed in NKT cell-deficient mice, we administered PBS or IL-4 complexed with an anti-IL4 antibody (IL-4c) to groups of CD1d^−/−^ mice during the initial 2 days of infection. Remarkably, administration of IL-4c for the initial days of infection is sufficient to restore a robust germinal center response and enhance IgG1 class-switching in the absence of NKT cells (Figures 7G and 7H). In contrast, administration of more antigen-specific TfH cell precursors to CD1d^−/−^ animals did not rescue the germinal center defect, indicating that the TfH cells might not compensate for the absence of NKT cells (Figures S7C–S7F). Altogether, our results indicate that the early wave of IL-4 triggered after influenza infection is critical for the induction of B cell responses.

Previous studies have shown that IL-4 supports the energy and biosynthetic needs necessary for growth, proliferation, and effector functions of immune cells (Dufort et al., 2007, Huang et al., 2016, Ricardo-Gonzalez et al., 2010). Thus, it is feasible that the early IL-4 wave would induce metabolic reprograming on antigen-experienced B cells to fulfil the high metabolic requirements associated with the generation of germinal centers. To test this hypothesis, we initially cultured murine naive B cells or B cells stimulated through their B cell receptor (BCR) (anti-IgM) in the presence or absence of IL-4. Interestingly, we observed that the addition of extracellular IL-4 to our *ex vivo* B cell cultures increases the oxygen consumption rate (OCR) in both naive and antigen-stimulated lymphocytes (Figure S7G). To address whether early IL-4 secretion could induce metabolic changes on B cells *in vivo*, we infected groups of mice with influenza virus and treated them with either PBS or anti-IL4 blocking antibody. We isolated lymph node cells on day 3 of infection, cultured them with anti-IgM and anti-CD3ε for 16 hr, and measured OCR levels in purified B cells after the incubation. We found that B cells from lung-draining lymph nodes have a higher OCR than B cells from non-draining lymph nodes, indicating that there is a metabolic reprograming of B cells early after influenza infection (Figure S7H). Interestingly, the ability of B cells to increase their respiratory capacity after viral infection requires early IL-4 production because the upregulation of the OCR is diminished when the early IL-4 wave is inhibited (Figure S7H). These results suggest that the early IL-4 wave triggered after infection could be regulating seeding of germinal center cells through the induction of metabolic changes in antigen-specific B cells.

### Early NKT Cell and IL-4 Signatures in Lymph Nodes from Zika-Infected Macaques

We were interested to find out whether the early role for NKT cells and IL-4 in the initiation of antiviral B cell immunity might extend to other species. To address this question, we analyzed transcriptomic data from lymph nodes of rhesus macaques that were subcutaneously infected with Zika virus (Aid et al., 2017). We assessed the transcriptomic profiles of lymph nodes obtained on day 3 or 14 post-infection to identify pathways that correlated with Zika-specific neutralizing antibody titers on day 7 and day 14, respectively (Figure 7I; Figure S7I). Pathway enrichment analysis revealed a significant and positive correlation of NKT cells (normalized enrichment score [NES] = 1.48, false discovery rate [FDR] = 0.06), IL-4 (NES = 1.43, FDR = 0.02), IL-18 (NES = 1.42, FDR = 0.05), and STAT6 signaling (NES = 1.83, FDR = 0.02) signatures as early as day 3 of infection with neutralizing antibody titers at day 7 (Figure 7J). Furthermore, gene-interacting network inference showed the high interconnectivity of all these pathways, highlighted by co-expression of their leading genes with a central role of IL-4 in connecting all of these genes (Figure 7K). Importantly, no significant correlation between TfH cell signatures (FDR > 0.20) and neutralizing antibodies was found at this early time point (Figure 7J). In contrast, a positive correlation between TfH cell signatures and neutralizing antibodies was observed on day 14 of infection (NES = 1.23, FDR = 0.13) (Figures S7J and S7K). These correlates are in line with our previous findings, pointing out to a possible role for NKT cells at the early stages of B cell immunity, with TfH cells only appearing later on the infection process. Furthermore, these analyses indicate that an early pre-TfH wave of NKT cells and IL-4 might be conserved upon influenza and Zika virus infection in mice and primates, respectively.

## Discussion

Several studies have focused on the use of NKT cell agonists as vaccine adjuvants to boost NKT cell activity and immune responses. In particular, immunization with inactivated influenza virus or virus-derived proteins in combination with α-GalCer, a synthetic glycosphingolipid, results in strong protection against subsequent challenges with various strains of influenza virus (Artiaga et al., 2016, Galli et al., 2007). However, whether NKT cells could play a role in the induction of adaptive immune responses in the absence of exogenous glycolipids was not clear. Here we showed that NKT cells are required for the early seeding of germinal center B cells and the production of class-switched antibody-secreting cells in response to influenza infection. Furthermore, we found that NKT cells can enhance B cell immunity to influenza-derived proteins when administered together with TLR adjuvants. Our findings are in line with previous studies showing that NKT cells enhance the activity of CD8^+^ T cells after influenza infection even in the absence of exogenous glycolipids (De Santo et al., 2008, Ishikawa et al., 2010). Therefore, the importance of NKT cells in the induction of adaptive immunity goes beyond the presence of glycolipid antigens.

How do NKT cells promote B cell responses during infection? Previous work showed that, in response to bacterium-derived glycolipids, NKT cells differentiate into NKTfH cells that engage in prolonged cognate interactions with B cells. These CD1d-mediated interactions rapidly induce germinal center and extrafollicular plasma cell formation, leading to the production of high titers of IgM and early class-switched antibodies (Bai et al., 2013, Barral et al., 2008, Chang et al., 2011, King et al., 2011, Leadbetter et al., 2008). However, we found that, upon influenza virus infection, NKT cells do not differentiate into NKTfH cells, nor they localize in germinal centers. Furthermore, CD1d-mediated cognate interactions between B cells and NKT cells are dispensable for the induction of antiviral B cell responses. Therefore, even though NKT cells promote B cell immunity in response to both bacterium-derived glycolipids and viral infection, the mechanism behind this regulation varies depending on the absence or presence of pathogen-derived glycolipids on the surface of the infectious agent.

Our results show that, during the initial stages of a viral infection, NKT cells become the main source of IL-4 in draining lymph nodes. Flow cytometry and single-cell RNA-seq analysis revealed that around 70% of the IL-4-producing cells are NKT cells, whereas the remaining ones are mainly T cells, excluding a significant contribution by ILC2s and TfH cells to the IL-4 production at early stages of infection. This finding was unexpected because previous studies reported that TfH cells constitute essentially all of the IL-4 producing cells in lymph nodes in response to infection with different pathogens (King and Mohrs, 2009, Reinhardt et al., 2009, Yusuf et al., 2010). However, this previously unreported NKT cell-mediated wave of IL-4 production occurs at the early stages of infection, even before the appearance of TfH cells or germinal centers. Furthermore, this phenomenon is restricted to the follicular borders, where B cells migrate immediately after pathogen encounter. The spatiotemporally restricted production of IL-4 by NKT cells appears to instruct antigen-activated B cells at the periphery of B cell follicles early after infection for the subsequent seeding of germinal centers. Interestingly, a recent report showed that TfH cells only switch to IL-4 production at the late stages of the infection process (Weinstein et al., 2016). Therefore, it will be important to discern the contribution of the early NKT cell IL-4 wave at the follicular borders versus the late TfH cell IL-4 wave at germinal centers to the different processes involved in the development of B cell immunity.

The positioning of NKT cells at the follicular borders during the early stages of infection appears to be at the core of the NKT cell ability to initiate B cell immunity. Single-cell RNA-seq and flow cytometry analysis revealed that IL-4-producing NKT cells preferentially express CXCR3, a chemokine receptor used by T cell subsets to relocate to the interfollicular areas of the lymph node (Groom et al., 2012, Sung et al., 2012). However, whether NKT cells acquire CXCR3 after activation or whether a population of NKT cells already expressing CXCR3 expands after infection remains to be elucidated. Interestingly, Th2 cells have also been shown to locate and exert their functions at the periphery of B cell follicles (Turqueti-Neves et al., 2014). Therefore, we believe our study reveals a new layer of regulation of B cell immunity at the initial stages of the infection process, when B cells relocate to follicular borders and recruit T cell help.

The early production of IL-4 by NKT cells is mainly triggered by CD169^+^ macrophages and is mediated by CD1d interactions and IL-18 secretion. Interestingly, a great number of NKT-macrophage contacts can be observed at the subcapsular sinus and interfollicular areas as early as 2 days post-influenza infection. Our findings are in line with the notion that NKT cell activation after infection is the result of a combination of both CD1d and cytokine signaling, with CD1d helping to position the cells and cytokines inducing the effector functions of NKT cells (Brennan et al., 2013). Furthermore, previous studies by others and us have shown that these macrophages can present bacterium-derived glycolipids on CD1d to mediate early NKT cell expansion and that IL-18 can enhance NKT cell activity in response to lipopolysaccharide (LPS), when co-administered with α-GalCer or in autoimmune models (Barral et al., 2010, Hägglöf et al., 2016, Leite-De-Moraes et al., 2001, Nagarajan and Kronenberg, 2007). Therefore, it seems that the induction of diverse NKT cell effector functions by CD169^+^ macrophages and IL-18 is a conserved mechanism observed upon infection with various pathogens and in autoimmune disorders.

In this study, we have unveiled a new layer of regulation of B cell immunity at the early stages of viral infection that involves a tightly regulated NKT cell-IL-4-B cell axis. Interestingly, this phenomenon seems to be conserved upon viral infection in other species because our transcriptomic analysis of Zika-virus-infected macaques revealed a positive correlation between early NKT cell-IL-4 gene signatures and the serum levels of Zika virus-neutralizing antibodies. We believe our findings raise the prospect of harnessing the interplay between macrophages and NKT cells and the early production of IL-4 at follicular borders in future approaches for the design of new vaccination strategies.

## STAR★Methods

### Key Resources Table

**Table:** 

REAGENT or RESOURCE	SOURCE	IDENTIFIER
**Antibodies**		

PE-Cy7 Rat Anti-Mouse CD19, Clone 1D3	ThermoFisher Scientific	Cat# 25-0193-82, RRID:AB_657663
Alexa Fluor 488 Mouse Anti-Mouse CD95 (APO-1/ Fas), Clone 15A7	ThermoFisher Scientific	Cat# 53-0951-82, RRID:AB_10671269
Alexa Fluor 647 Rat Anti-Mouse/Human GL-7 (T and B cell activation Marker), Clone GL7	Biolegend	Cat# 144606, RRID:AB_2562185
PE Rat Anti-Mouse CD4, Clone GK1.5	Biolegend	Cat# 100408, RRID:AB_312693
APC Rat Anti-Mouse CD4, Clone GK1.5	Biolegend	Cat# 100412, RRID:AB_312697
Biotin Rat Anti-Mouse CD185 (CXCR5), Clone SPRCL5	ThermoFisher Scientific	Cat# 13-7185-82, RRID:AB_2572800
PerCP eFluor 710 Rat Anti-Mouse CD185 (CXCR5), Clone SPRCL5	ThermoFisher Scientific	Cat# 46-7185-82, RRID:AB_2573837
FITC Hamster Anti-Mouse CD279 (PD-1), Clone J43	ThermoFisher Scientific	Cat# 11-9985-82, RRID:AB_465472
Pacific Blue Rat Anti-Mouse/Human CD45R (B220), Clone RA3-6B2	Biolegend	Cat# 103227, RRID:AB_492876
Brilliant Violet 650 Rat Anti-Mouse CD45R (B220), Clone RA3-6B2	Biolegend	Cat# 103241, RRID:AB_11204069
APC Rat Anti-Mouse MHC Class II (I-A/I-E), Clone M5/114.15.2	ThermoFisher Scientific	Cat# 17-5321-82, RRID:AB_469455
PerCP/Cy5.5 Armenian Hamster Anti-Mouse CD11c, Clone N418	Biolegend	Cat# 117328, RRID:AB_2129641
eFluor 450 Armenian Hamster Anti-Mouse CD11c, Clone N418	ThermoFisher Scientific	Cat# 48-0114-82, RRID:AB_1548654
APC Rat Anti-Mouse CD11b, Clone M1/70	BD Biosciences	Cat# 553312, RRID:AB_398535
eFluor 450 Rat Anti-Mouse CD11b, Clone M1/70	ThermoFisher Scientific	Cat# 48-0112-82, RRID:AB_1582236
eFluor 450 Rat Anti-Mouse CD3, Clone 17A2	ThermoFisher Scientific	Cat# 48-0032-82, RRID:AB_1272193
eFluor 450 Rat Anti-Mouse CD5, Clone 53-7.3	ThermoFisher Scientific	Cat# 48-0051-82, RRID:AB_1603250
PE Rat Anti-Mouse CD127 (IL-7Rα), Clone A7R34	Biolegend	Cat# 135010, RRID:AB_1937251
FITC Armenian Hamster Anti-Mouse TCRβ, Clone H57-597	ThermoFisher Scientific	Cat# 11-5961-82 RRID:AB_465323
APC Armenian Hamster Anti-Mouse TCRβ, Clone H57-597	ThermoFisher Scientific	Cat# 17-5961-82 RRID:AB_469481
PE PBS57-loaded Mouse CD1d tetramer	NIH Tetramer Core Facility	N/A
PE-Cy7 Armenian Hamster Anti-Mouse CD69, Clone H1.2F3	ThermoFisher Scientific	Cat# 25-0691-82, RRID:AB_469637
PE Rat anti-Mouse CD1d, Clone 1B1	BD Biosciences	Cat# 553846, RRID:AB_2073521
Pacific Blue Rat Anti-Mouse Ly6G-Ly6C (Gr-1), Clone RB6-8C5	Biolegend	Cat# 108430, RRID:AB_893556
V450 Rat Anti-Mouse IgG1, Clone A85-1	BD Biosciences	Cat# 562107, RRID:AB_10894002
PerCP/Cy5.5 Mouse anti-Mouse CD45.1, Clone A20	ThermoFisher Scientific	Cat# 45-0453-82, RRID:AB_1107003
Pacific Blue Mouse anti-Mouse CD45.2, Clone 104	Biolegend	Cat# 109820, RRID:AB_492872
PerCP/Cy5.5 Rat anti-Mouse CD45, Clone 30-F11	Biolegend	Cat# 103132, RRID:AB_893340
PE-Cy7 Armenian Hamster Anti-Mouse CD183 (CXCR3), Clone CXCR3-173	Biolegend	Cat# 126516, RRID:AB_2245493
APC Armenian Hamster Anti-Mouse CD183 (CXCR3), Clone CXCR3-173	Biolegend	Cat# 126512, RRID:AB_1088993
Alexa Fluor 488 Rat Anti-Mouse CD150 (SLAM), Clone TC15-12F12.2	Biolegend	Cat# 115916, RRID:AB_528744
APC Rat Anti-Mouse CD8a, Clone 53-6.7	Biolegend	Cat# 100712, RRID:AB_312751
Purified Rat Anti-Mouse CD16/32, Clone 93	Biolegend	Cat# 101302, RRID:AB_312801
Alexa Fluor 647 Mouse Anti-Mouse GATA-3, Clone 16E10A23	Biolegend	Cat# 653810, RRID:AB_2563217
APC Rat Anti-Mouse RORγt, Clone B2D	ThermoFisher Scientific	Cat# 17-6981-82, RRID:AB_2573254
PE-Cy7 Mouse Anti-Mouse T-bet, Clone 4B10	ThermoFisher Scientific	Cat# 25-5825-82, RRID:AB_11042699
Alexa Fluor 647 Mouse Anti-Mouse Bcl-6, Clone K112-91	BD Biosciences	Cat# 561525, RRID:AB_10898007
PE-Cy7 Rat Anti-Mouse IFN-γ, Clone XMG1.2	ThermoFisher Scientific	Cat# 25-7311-41, RRID:AB_1257211
APC Rat Anti-Mouse IL-4, Clone 11B11	ThermoFisher Scientific	Cat# 17-7041-82, RRID:AB_469494
InVivoMab Rat Anti-Mouse IL-4, Clone 11B11	BioXCell	Cat# BE0045, RRID:AB_1107707
Alexa Fluor 488 Rat Anti-Mouse CD169 (Siglec-1), Clone SER-4	Produced in the lab	N/A
FITC Rat Anti-Mouse IgD, Clone 11-26c.2a	BD Biosciences	Cat# 553439, RRID:AB_394859
Alexa Fluor 647 Rat Anti-Mouse CD207 (Langerin), Clone 929F3.01	Dendritics	Cat# DDX0362, RRID:AB_1148742
Purified Goat Anti-Mouse IL-1β, Polyclonal	R&D Systems	Cat# AF-401-NA, RRID:AB_416684
Purified Rabbit Anti-Mouse IL-18, Polyclonal	Abcam	Cat# ab71495, RRID:AB_1209302
Purified Goat Anti-Mouse IL-33, Polyclonal	R&D Systems	Cat# AF3626, RRID:AB_884269
Alexa Fluor 555 Goat anti-Rabbit IgG (H + L)	ThermoFisher Scientific	Cat# A32732 RRID:AB_2633281
Alexa Fluor 555 Donkey anti-Goat IgG (H + L)	ThermoFisher Scientific	Cat# A-21432, RRID:AB_141788
Purified Goat anti-R-Phycoerythrin, Polyclonal	Novus Biologicals	Cat# NB100-78573, RRID:AB_1085348
Biotin Goat Anti-Mouse IgM, Human adsorbed	Southern Biotech	Cat# 102008, RRID:AB_616726
Biotin Goat Anti-Mouse IgG1, Human adsorbed	Southern Biotech	Cat# 107008, RRID:AB_609714
Purified Armenian Hamster anti-Mouse CD3ε, Clone 145-2C11	Biolegend	Cat# 100302, RRID:AB_312667

**Bacterial and Virus Strains**		

Influenza virus A/Puerto Rico/8/1934 (PR8) H1N1	Caetano Reis e Sousa’s lab	N/A
PR8-OTII	Thomas et al., 2006	N/A
Influenza NS1-GFP	Manicassamy et al., 2010	N/A
Vaccinia virus Western Reserve	Caetano Reis e Sousa’s lab	N/A

**Chemicals, Peptides, and Recombinant Proteins**		

PE Streptavidin	Biolegend	Cat# 405204
eFluor 450 Streptavidin	ThermoFisher Scientific	Cat# 48-4317
PR8 Hemagglutinin trimer	From Daniel Lingwood	N/A
Poly I:C HMW	Invivogen	Cat# 31852-29-6
Alpha Gal-Cer KRN7000	Enzo Life Sciences	Cat# BML-SL232
Diphtheria Toxin from Corynebacterium diphtheriae	Sigma	Cat# D0564
Recombinant Murine Interleukin 4	Prepotech	Cat# 214-14
LIVE/DEAD Fixable Blue	ThermoFisher Scientific	Cat# L23105
Cell Stimulation Cocktail plus protein transport inhibitor	ThermoFisher Scientific	Cat# 00-4975-03
Influenza A H1N1 (A/Puerto Rico/8/34) Hemagglutinin	Sino Biological	Cat# 11684-V08H
Streptavidin-Alkaline Phosphatase	Sigma	Cat# S2890
Recombinant Mouse Interleukin 18	R&D Systems	Cat# 9139-IL
AffiniPure F(ab’)_2_ Fragment Goat Anti-Mouse IgM, μ Chain Specific	Jackson ImmunoResearch	Cat# 115-006-020
3-Amino-9-ethylcarbazole	Sigma	Cat# A5754

**Critical Commercial Assays**		

Foxp3 Transcription Factor Staining Buffer Set	ThermoFisher Scientific	Cat# 00-5523-00
Cytofix/Cytoperm	BD Biosciences	Cat# 554722
RNeasy Micro Kit	QIAGEN	Cat# 74004
SuperScript III first-strand system	ThermoFisher Scientific	Cat# 18080051
SYBR green PCR master mix	ThermoFisher Scientific	Cat# 4309155
Mouse IL-4 ELISPOT kit	BD Biosciences	Cat# 551017
Pan B cell Isolation Kit, mouse	Miltenyi	Cat# 130-095-813
Seahorse XF Cell Mito Stress Test Kit	Agilent	Cat# 103015-100
Maxima Reverse Transcriptase	ThermoFisher Scientific	Cat# EP0741

**Deposited Data**		

Single-Cell RNA-Seq data	This study	GEO: GSE103753

**Experimental Models: Cell Lines**		

Dog (female), MDCK cells	Caetano Reis e Sousa’s lab	N/A
Chicken, DF-1 cells	Caetano Reis e Sousa’s lab	N/A

**Experimental Models: Organisms/Strains**		

Mouse: *Wild type* (C57BL/6J)	The Jackson Laboratory	Strain Code: JAX 000664
Mouse: *Wild type* (FVB/NCrl)	Charles Rivers Laboratory	Strain Code: 207
Mouse: *B6.SJL-Ptprc*^*a*^*Pepc*^*b*^*/BoyJ* (CD45.1)	The Jackson Laboratory	Strain Code: JAX 002014
Mouse: *B6;129-Siglec1*^*< tm1(HBEGF)Mtka >*^ (CD169-DTR)	Riken BioResource Center	Strain Code: RBRC04395
Mouse, C.Cg-Cd19^tm1(cre)Cgn^ Igh^b^/J (CD19-Cre)	The Jackson Laboratory	Strain Code: JAX 004126
Mouse, B6.129S6-Del(3Cd1d2-Cd1d1)1Sbp/J (CD1d KO)	The Jackson Laboratory	Strain Code: JAX 008881
Mouse, B6.129S7-Il1r1^tm1Imx^/J (IL-1R KO)	The Jackson Laboratory	Strain Code: JAX 003245
Mouse, B6.129P2-Il18r1^tm1Aki^/J (IL-18R KO)	The Jackson Laboratory	Strain Code: JAX 004131
Mouse, B6.129P2-Lyz2tm1(cre)Ifo/J (Lyz2-Cre)	The Jackson Laboratory	Strain Code: JAX 004781
Mouse, B6.Cg-Tg(Itgax-cre)1-1Reiz/J (CD11c-Cre)	The Jackson Laboratory	Strain Code: JAX 008068
Mouse, B6.129S1-Tlr7^tm1Flv^/J (TLR7 KO)	The Jackson Laboratory	Strain Code: JAX 008380
Mouse, C57BL/6-Tg(TcraTcrb)425Cbn/Crl (OT-II)	The Jackson Laboratory	Strain Code: JAX 004194
Mouse, B6.129P2(SJL)-Myd88^tm1.1Defr^/J (MyD88 KO)	The Jackson Laboratory	Strain Code: JAX 009088
Mouse, C.129-Il4^tm1Lky^/J (IL-4/GFP-enhanced transcript, 4Get). Backcrossed to B6 background for 10 generations.	The Jackson Laboratory	Strain Code: JAX 004190
Mouse, B6.C(Cg)-Cd79a^tm1(cre)Reth^/EhobJ (Mb1-Cre)	The Jackson Laboratory	Strain Code: JAX 020505
Mouse, B6.129P2-Il4^tm1Cgn^/J (IL-4 KO). Backcrossed to FVB/N background for 10 generations.	The Jackson Laboratory	Strain Code: JAX 002253
Mouse, Il1rl1^tm1Anjm^ (IL-33R KO)	Townsend et al., 2000	MGI: 2386675
Mouse, CD1d ^flox/flox^ CD19-Cre	This study	N/A
Mouse, CD1d ^flox/flox^ Mb1-Cre	This study	N/A
Mouse, CD1d ^flox/flox^ Lyz2-Cre	This study	N/A
Mouse, CD1d ^flox/flox^ CD11c-Cre	This study	N/A
Mouse, B6.Cg-Tg(Pgk1-flpo)10Sykr/J (FLPo)	The Jackson Laboratory	Strain Code: JAX 011065

**Oligonucleotides**		

Primer: *HPRT* Forward GCCCTTGACTATAATGAGTACTTCAGG	This study	N/A
Primer: *HPRT* Reverse TTCAACTTGCGCTCATCTTAGG	This study	N/A
Primer: *IL-4* Forward ACGAGGTCACAGGAGAAGGGA	This study	N/A
Primer: *IL-4* Reverse AGCCCTACAGACGAGCTCACTC	This study	N/A
Primer: *IL-5* Forward TGACAAGCAATGAGACGATGAGG	This study	N/A
Primer: *IL-5* Reverse ACCCCCACGGACAGTTTGATTC	This study	N/A
Primer: *IL-13* Forward CCTCTGACCCTTAAGGAGCTTAT	This study	N/A
Primer: *IL-13* Reverse CGTTGCACAGGGGAGTCTT	This study	N/A
Primer: *IL-21* Forward TCAGCTCCACAAGATGTAAAGGG	This study	N/A
Primer: *IL-21* Reverse GGGCCACGAGGTCAATGAT	This study	N/A

**Software and Algorithms**		

Diva	BD Biosciences	http://www.bdbiosciences.com/us/instruments/clinical/software/flow-cytometry-acquisition/bd-facsdiva-software/m/333333/overview
FlowJo (version 10.1)	FlowJo, LLC	https://www.flowjo.com
Zen	Zeizz	https://www.zeiss.com/microscopy/int/downloads/zen.html
ImageJ (version 1.50c)	NIH Image	https://imagej.nih.gov/ij/
Imaris (version 8.2.0)	Bitplane	http://www.bitplane.com/
GraphPad Prism (version 7.0)	GraphPad Software Inc	https://www.graphpad.com/scientific-software/prism/
ELISPOT reader with immunospot software	Cellular Technology Ltd	N/A
InDesign CS5.5	Adobe	http://www.adobe.com/products/indesign.html
R (Version 3.3.2), Seurat package (version 1.4.0.16)	R Core Team	https://www.r-project.org/
GSEA	Broad Institute	http://software.broadinstitute.org/gsea/index.jsp
Gene functional annotation and network inference	http://cytoscape.org/	http://genemania.org/

### Contact for Reagent and Resource Sharing

Further information and requests for resources and reagents should be directed to, and will be fulfilled by, the Lead Contact, Facundo D. Batista (FBATISTA1@mgh.harvard.edu).

### Experimental Model and Subject Details

#### Mice

8-10 week old wild-type C57BL/6 and FVB/N mice were obtained from Jackson and Charles River Laboratories. Congenic CD45.1 mice were from the Francis Crick Institute, UK. Siglec1^DTR/+^ (CD169-DTR) mice were obtained from the Riken BioResource Center, Japan. CD19-Cre, CD1d^−/−^, IL-1R^−/−^ and IL-18R^−/−^ mice were obtained from Jackson Laboratories, USA. Lyz2-Cre, Cd11c-Cre, TLR7^−/−^, OT-II and Myd88^−/−^ mice were obtained from Dr. Caetano Reis e Sousa, Francis Crick Institute, UK. IL-4/GFP-enhanced transcript (4Get) mice were obtained from Dr. Mark Wilson, Francis Crick Institute, UK. FVB/N IL-4^−/−^ mice were obtained from Dr. Jessica Strid, Imperial College London, UK. Mb1-Cre mice were obtained from Dr. Michael Reth, Max Planck Institute, Germany. IL-33R^−/−^ bone marrows (Townsend et al., 2000) were obtained from Andrew McKenzie, MRC laboratory of Molecular Biology, UK and Padraic Fallon, Trinity College Dublin, Ireland.

For the generation of CD1d conditional mice, we used homologous recombination to insert *loxP* sites flanking exon 3 of *cd1d1 gene* in B6 embryonic stem (ES) cells. Identification of ES cell clones with a single correct insertion of the targeted locus in the genome was performed through long-range PCR and Southern blot. Transgenic mice derived from these ES cells were crossed with B6 mice expressing the FLP recombinase to remove the *frt*-flanked *PGK-neomycin* sequence used for selection of ES cells that have inserted the targeted vector into their genome. Mice carrying the *flox* allele were crossed with mice expressing the Cre recombinase under the promoters of CD19, Mb1, Lyz2 and CD11c genes. Finally, mice were crossed again with the same floxed mice to make them homozygous for the floxed allele.

For the generation of mixed bone marrow chimeras, CD45.1 C57BL/6 or CD169-DTR of 6-8 weeks of age were irradiated with 2 doses of 500 rad, 4 hours apart. One day later, bone marrow cells were injected i.v. in recipient animals (2x10^6^ cells in total). Experimental animals were kept on acidified water for one week prior and 3 weeks post irradiation treatment. Chimeras were used after 8 weeks of reconstitution.

Mice were bred and maintained at the animal facilities of the Francis Crick and Ragon Institutes. All mice were maintained in individually ventilated cages and were used at the age of 8 to 12 weeks. Up to five mice per cage were housed in individually ventilated cages in a 12 hr light/dark cycle, with room temperature at 22 C (19 C-23 C change). They were fed with autoclaved standard pellet chow and reverse osmosis water. All cages contained 5 mm of aspen chip and tissue wiles for bedding and a mouse house for environmental enrichment. Littermates (males or females) were randomly assigned to experimental groups. Generally between 4 to 8 mice were used per experimental group. All experiments were approved by the Animal Ethics Committee of Cancer Research UK and the United Kingdom Home Office, and by the Institutional Animal Care and Use Committee of the United States.

#### Infections and injections

Mice were anesthetized i.p. with Ketamine/Xylazine (100 mg/kg body) and intranasally infected with 200 PFU of Influenza virus A/Puerto Rico/8/1934 (PR8) H1N1 strain or 100 PFU of PR8-OTII virus (Thomas et al., 2006) or 10^3^ PFU of Influenza NS1-GFP virus (Manicassamy et al., 2010) or 200 PFU of Vaccinia virus Western Reserve strain in 20 μl of PBS. Influenza virus was amplified on MDCK cells and VACV on DF-1 cells. Purification of viral particles was performed in a sucrose 30% cushion at 25, 000 RPM for 2 hours in an SW32Ti rotor. For protein immunizations, 5 μg of PR8 Hemagglutinin (HA) trimmer, kindly provided by Daniel Lingwood, was intranasally administered together with 10 μg of Poly I:C (Invivogen) or 2 μg of α-GalCer (Enzo) in 20 μl of PBS. For depletion of CD169^+^ macrophages, mice were i.p. injected with 500 ng of Diphtheria toxin (Sigma) in 200 μl of PBS. For *in vivo* IL-4 blocking, mice were i.p. injected with 1.5 mg of anti-IL4 (11B11, BioXCell) in 200 μl of PBS at different days of infection. For the administration of IL-4 complex (IL-4c), 5 μg of recombinant mouse IL-4 (PrepoTech) was complexed to 25 μg of anti-mouse IL-4 (BioXCell, 11B11), diluted in 200 μl of PBS and administered i.p. on days 0, 1 and 2 of infection. For adoptive transfers, sorted NKT cells (8.10^5^), OT-II CD4^+^ isolated CD4^+^ T cells (2.10^4^) or bone marrow cells (2.10^6^) were resuspended in 200 μl of PBS and injected in the tail vein.

#### Flow Cytometry

For labeling of surface markers, single cell suspensions from lymph nodes or lungs were incubated with anti-mouse CD16/32 (BD Biosciences) and LIVE/DEAD Fixable blue (Life Technologies) in PBS 2% FCS for 20 minutes on ice to block nonspecific antibody binding and exclude dead cells. Cells were then washed and stained for 20 minutes on ice with the indicated anti-mouse antibodies, PBS57-loaded CD1d tetramer (kindly provided by NIH tetramer core facility) and/or biotinylated HA (Sino biological). When using biotinylated antibodies or proteins, cells suspensions were washed following antibody labeling and incubated for 20 minutes on ice with labeled streptavidin. If labeling of transcription factors was performed, cells were fixed and permeabilized with Foxp3 transcription factor staining buffer set (eBioscience) according to manufacturer instructions and incubated for 30 min with anti-mouse antibodies. Finally, cells were either resuspended in 400μl of FACS buffer and analyzed on a Fortessa cytometer (BD Biosciences) or in RPMI media and used for cell sorting on a FACSAria II (BD, Bioscience).

For analysis of cytokine production, lymph node single cell suspensions were incubated 4 hours at 37°C in RPMI complete medium supplemented with 1X Cell stimulation cocktail plus protein transport inhibitor (eBioscience). After surface staining, cells were fixed and permeabilized with Cytofix/Cytoperm (BD Biosciences) according to manufacturer instructions and incubated for 30 min with anti-mouse antibodies. Finally, cells were resuspended in 400μl of FACS buffer and data were collected on a Fortessa cytometer (BD Biosciences) and analyzed with FlowJo (TreeStar).

#### Immunohistochemistry

For surface staining, cryostat sections (10μm thick) of lymph nodes were dried, fixed in 4% PFA for 10 minutes, blocked with PBS 5% BSA and then incubated with the following anti-mouse antibodies in 1% BSA for 1 hour: B220 Pacific Blue (RA3-6 B2, Biolegend), CD169 AF488 (Ser-4, ATCC), IgD FITC (11-26c.2a, BD Biosciences), Langerin AF647 (929F3.01, Dendritics) and GL-7 AF647 (GL7, Biolegend). For intracellular staining, sections were permeabilized with PBS Triton 0.3% for 5 minutes before blocking and then incubated with anti-mouse IL-1β (polyclonal, R&D), IL-18 (polyclonal, Abcam) or IL-33 (polyclonal, R&D), followed by incubation with AF555 anti-goat or anti-rabbit IgG (Invitrogen). CD1d tetramer staining was performed as previously described (Lee et al., 2015). Briefly, lymph nodes were incubated overnight with PE-labeled PBS-57 loaded CD1d tetramer in 2% FCS at 4°C. Next day, lymph nodes were washed and fixed with 4% PFA for 1 hour and snap frozen. 10μm sections were blocked with 5% BSA and stained with anti-PE antibody (polyclonal, Novus Biologicals) followed by goat anti-rabbit AF555 (Life Technology). Imaging was carried out on a LSM 780 (Zeiss) inverted confocal microscope using a Plan-Apochromat 40x NA 1.3 oil immersion objective.

#### qRT-PCR

NKT cells, TfH cells and conventional CD4^+^ T cells from mediastinal lymph nodes were sorted at day 3 and 9 of infection using the gating strategy shown in Figure S2A. RNA was extracted from sorted cells using the RNeasy micro Kit (QIAGEN). Between 10^3^ and 10^4^ cells were used in each condition. mRNA was reversely transcribed into cDNA using random hexamers from the SuperScript III first-strand system (Thermofisher). cDNA was amplified using custom primer sets (Sigma) and SYBR green PCR master mix (Thermofisher) on a 7500 real-time PCR system (Applied Biosystems). The relative mRNA abundance of target genes was determined by means of the ΔΔCT method, using HPRT as house keeping gene.

#### ELISPOT

To measure influenza-specific ASCs, Enzyme-linked immunosorbent spot (ELISPOT) multiscreen filtration plates (Millipore) were activated with absolute ethanol, washed with PBS and coated overnight at 4°C with 1 μg/ml of PR8 virus or 2 μg/ml of PR8 HA (Sino Biological) diluted in PBS. Plates were subsequently blocked for 1 hour with complete medium and incubated for 24 hours at 37°C with serial dilutions of lymph node single cell suspensions. Plates were washed with PBS 0.01% Tween-20 and incubated for 1 hour with 1μg/ml of biotinylated anti-IgM or IgG1 (Southern Biotech) diluted in PBS 1% BSA. Then, plates were washed and incubated for 1 hour with 1μg/ml Streptavidin-Alkaline Phosphatase (Sigma). Finally plates were washed and developed with 3-amino-9-ethyl-carbazole (Sigma).

To measure IL-4 production after influenza infection, NKT cells were sorted from mediastinal lymph nodes (TCRβ^+^ CD1d-tetramer^+^ CXCR3^+/−^) and incubated in RPMI complete medium supplemented with 1X Cell stimulation cocktail (eBioscience) for 32 hours at a density of 2.10^4^ cells/well. To measure IL-4 production in the presence of IL-18, NKT cells were sorted from spleen (TCRβ^+^ CD1d-tetramer^+^) and incubated with increasing concentrations of mouse recombinant IL-18 (R&D systems) for 32 hours at a density of 2.10^4^ cells/well. IL-4 secreting cells were detected using the mouse IL-4 ELISPOT kit from BD Biosciences following manufacturer’s instructions.

#### Extracellular flux assay

Splenic naive B cells were purified using Pan B cell Isolation Kit (Miltenyi) and stimulated with 10 ng/ml IL-4 (PrepoTech) and/or 5 μg/ml F(ab’)_2_ anti-IgM (Jackson ImmunoResearch) for 16 hours. Lymph node cells were obtained after 3 days of influenza infection, cultured with 5 μg/ml F(ab’)_2_ anti-IgM and 3 μg/ml anti-CD3ε (Biolegend, 145-2C11) and B cells were purified after 16 hours. B cells were resuspended in Seahorse medium supplemented with 11mM glucose (Sigma Aldrich), 2 mM glutamax (Life Technologies) and 1 mM Sodium pyruvate (Life Technologies). Cells were settled on 96-well assay plate (Seahorse Bioscience) coated with poly-lysine (Sigma Aldrich) for 30 minutes. Data was recorded with the XF96 Extracellular Flux analyzer. Oligomycin A (3 μM), FCCP (5 μM) and Rotenone (5 μM) (all from Agilent) were added sequentially at indicated time points to generate the OCR profile.

#### scRNA-Seq Library Preparation

Plate-based scRNA-Seq libraries were prepared as previously described (Trombetta et al., 2014). Briefly, cells were FACS sorted into 96-well plates containing 10 μL of RLT lysis buffer (QIAGEN) supplemented with 1% β-mercaptoethanol (Aldrich) in each well. Two plates of GFP^+^CD1d-tetramer^+^ and two plates of the GFP^+^CD1d-tetramer^-^ cells were sorted. Smart-Seq2 whole transcriptome amplification (WTA) was performed as per (Trombetta et al., 2014) with Maxima Reverse Transcriptase used in place of SuperScript II (Life Technologies). Libraries were sequenced on an Illumina NextSeq500, with 30 base-pair paired-end reads, to an average depth of 670k ± 30k reads per cell.

#### Single-cell RNA-seq Data Analysis

Sequenced reads were aligned to the UCSC mm10 mouse transcriptome and gene expression was calculated using RSEM as previously described (Tirosh et al., 2016). Cells with greater than 1,000 genes mapped and with more than 10,000 transcripts detected were used for downstream phenotypic analysis. Genes expressed in fewer than 15 cells were filtered out from further analysis, leaving 222 cells and 9,271 genes. Expression data was log transformed (Log2(tpm+1)) before further analysis. Clustering and differential expression were performed using the Seurat package (version 1.4.0.16) in R (version 3.3.2) (Satija et al., 2015). Briefly, we identified 708 variable genes with log-mean expression values greater than 0.05 and dispersion (variance/mean) greater than 0.8. A principal components analysis was then run over variable genes, and the first five principal components were selected for downstream analyses based on a combination of Q-Q plots and the standard deviation of PCs as determined by an elbow plot (calculated using the Jackstraw algorithm in Seurat). tSNE and SNN clustering (FindClusters function in Seurat with k.param = 20 & resolution = 0.6) were used to depict and cluster the cells in each analysis, respectively. Genes differentially expressed between identified clusters were calculated using the bimodal distribution option. Differentially expressed markers were removed if identified in less than 20 percent of cells in a given cluster or if the average log fold change between clusters was less than 0.5. Heatmaps depict the 25 most significantly differentially expressed genes (p values < 0.002) in each cluster (full data available in Table S1). Violin plots were generated over curated NKT genes.

#### Transcriptomic analysis of samples from Zika infected macaques

Genes signatures for NKT, NKT subpopulations, IL4, IL18, TfH, STAT6 and B cell signaling were compiled from different public repository (http://software.broadinstitute.org/gsea/msigdb/, http://pathcards.genecards.org/, PMID:16698943, PMCID:PMC3528072). We correlated the expression of these signatures in lymph nodes from ZIKA virus infected Rhesus monkeys to neutralizing antibodies measured in plasma from the same animals on day 7 and day 14 (Aid et al., 2017). We used a linear regression analysis (R language) to identify genes that correlated significantly to neutralizing antibodies or viral loads using a p value cut-off of 0.05. Significant genes were then used to perform a genesets enrichment analysis using the above signatures (http://software.broadinstitute.org/gsea/index.jsp). Significant genes were used to infer gene interacting networks using GeneMania (http://genemania.org/) and Cytoscape (http://cytoscape.org/).

### Quantification and Statistical Analysis

Statistical parameters including the exact value of n with the description of what n represents, the mean, the SEM and the p value are reported in the Figures and the Figure Legends. Statistical analyses were performed using Prism (GraphPad Software), two-tailed t test, p values < 0.05 were considered significant. In figures, asterisks stand for: ^∗^, p < 0.05; ^∗∗^, p < 0.01; ^∗∗∗^, p < 0.001; ^∗∗∗∗^, p < 0.0001).

### Data and Software Availability

The single-cell RNA-seq data reported in this paper have been deposited in the NCBI Gene Expression Omnibus (GEO) database under accession number GEO: GSE103753.

## Author Contributions

M.G. designed and performed experiments, generated CD1d-floxed mice, analyzed the data, and wrote the manuscript. P.B. performed some initial experiments, assist with the setup of *in vivo* infections, and contributed to scientific discussions. A.W.N. and A.K.S. performed and analyzed single-cell RNA-seq data. M.B., C.T., S.A., and P.M. performed experiments and contributed to scientific discussions. M.A. and D.H.B. analyzed transcriptomic data from Zika-virus-infected macaques and contributed to scientific discussions. K.G. and R.-P.S. contributed to scientific discussions regarding transcriptomic analyses. P.G.F. provided IL-33R^−/−^ bone marrow. B.M. contributed to the generation of CD1d-floxed animals and provided technical support. A.B. analyzed data and provided technical support for microscopy. U.N. edited the manuscript. J.S. provided IL-4^−/−^ mice and contributed to scientific discussions. F.D.B. conceived the project, supervised the work, and wrote the manuscript.

## Supplemental Information

Table S1. List of Differentially Expressed Genes Identified by Single-Cell RNA-Seq among the IL-4+ Clusters, Related to Figure 4
